# Silencing the Signal: The Metastasis Suppressor NDRG1 Disrupts Small Extracellular Vesicle‐Mediated Crosstalk in Pancreatic Cancer

**DOI:** 10.1002/jev2.70334

**Published:** 2026-06-30

**Authors:** Jiawei Chang, Shafi Alenizi, Heloisa Zaccaron Milioli, Winston Lay, Saranya Pounraj, Yujie Li, Mekonnen Sisay Shiferaw, Elham Hosseini‐Beheshti, Zaklina Kovacevic

**Affiliations:** ^1^ Department of Physiology School of Biomedical Sciences Faculty of Medicine and Health University of NSW Sydney Australia; ^2^ School of Medical Sciences Faculty of Medicine and Health The University of Sydney Sydney Australia; ^3^ Garvan Institute of Medical Research Darlinghurst New South Wales Australia; ^4^ Asbestos and Dust Disease Research Institute (ADDRI) Sydney New South Wales Australia

**Keywords:** ALIX, ESCRT pathway, NDRG1, pancreatic cancer, sEVs, tumour microenvironment

## Abstract

Pancreatic cancer (PaC) remains one of the deadliest cancers, with 5‐year survival rates of 13%. A major driver of its aggressiveness is the tumour microenvironment (TME), which fuels tumour growth, metastasis, and therapeutic resistance through dynamic, bi‐directional communication between cancer cells, fibroblasts, and immune cells. Emerging evidence highlights extracellular vesicles (EVs) as key mediators of oncogenic cross‐talk within the PaC TME. This study demonstrates for the first time that the overexpression of metastasis suppressor N‐myc downstream regulated gene 1 (NDRG1) significantly influences the biogenesis, cargo packaging and release of EVs by cancer cells. This was mediated by a direct interaction between NDRG1 and ALIX, a key protein involved in EV biogenesis and packaging, with NDRG1 facilitating ALIX proteasomal degradation. Further, EVs released from NDRG1‐overexpressing cells had significantly fewer CAF‐activation proteins (i.e. TGF‐β), leading to attenuated ERK1/2 and p38 activation in pancreatic stellate cells (PSCs), and reduced expression of key fibrotic markers (α‐SMA, FAP, and collagen 1A). NDRG1 overexpression also reduced sEVs uptake by PaC cells and diverted these to the lysosome for degradation. These findings uncover a previously unrecognized mechanism by which NDRG1 overexpression disrupts the oncogenic two‐way communication between PaC cells and the TME, positioning NDRG1 overexpression as a compelling therapeutic approach against this formidable malignancy.

## Graphical abstract

NDRG1 suppresses pancreatic cancer progression by disrupting small extracellular vesicle biogenesis, altering cargo composition, and inhibiting EV uptake by cancer cells—thereby impairing tumour–stroma communication and fibrotic activation. This study reveals NDRG1 as a novel regulator of extracellular vesicle dynamics and a promising therapeutic target.

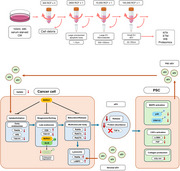

## 1 Introduction

Pancreatic cancer (PaC) is a highly aggressive malignancy associated with very poor 5‐year survival rates of 13%. Current PaC treatments fail to significantly extend survival due to limited drug penetration into the hypovascularized and dense PaC tumour microenvironment (TME) (Neesse et al. 2011). Over the past decade, extracellular vesicles (EVs) have emerged as critical mediators of intercellular communication within the cancer TME. These nanosized membrane‐encapsulated vesicles contain bioactive materials such as nucleic acids, fatty acids, proteins, and metabolites that are secreted by almost all cell types (Théry et al. 2009). Cancer cells release EVs through different biogenesis pathways to interact with cells within the TME and extracellular matrix (ECM), creating a favourable niche for proliferation and invasion (Moeng et al. 2020). EVs interact with other cells in local and distant locations, potentially altering the activity of recipient cells (Valadi et al. 2007). In PaC, EVs were found to facilitate cancer cell migration and invasion (Quail and Joyce 2013) and to prepare the pre‐metastatic niche at distal sites such as the liver (Li et al. 2025). Mounting evidence suggests that the oncogenic progression of PaC is tightly associated with cellular alterations caused by EVs (Moeng et al. 2020; Ren et al. 2018).

Bidirectional communication between cancer and stromal cells via EVs drives multiple aspects of PaC progression. Pancreatic stellate cells (PSCs), which are abundant in the pancreas and a key pre‐cursor to cancer‐associated fibroblasts (CAFs; [Manoukian et al. 2021]), release EVs containing miRNA cargo that promote PaC cell proliferation, migration, and metastasis in both cell and animal models (Kong et al. 2019; Pessolano et al. 2018; Li et al. 2020; Li et al. 2018; Yin et al. 2020), while also contributing to gemcitabine resistance (Fang et al. 2019; Mikamori et al. 2017). Conversely, PaC cell‐derived EVs alter the function of neighbouring CAF and immune cells in the TME to promote desmoplasia and immune escape (Liu et al. 2025; Purushothaman et al. 2023). These EVs contain bioactive materials that can initiate the activation of PSCs into CAFs, promote CAF collagen production, and polarize local macrophages into the oncogenic M2 phenotype (Moeng et al. 2020; Mikamori et al. 2017).

Small EVs (sEVs), the most extensively studied subtype of EVs, undergo a tightly regulated biogenesis process involving two main regulatory pathways: the endosomal sorting complex required for transport (ESCRT) pathway and small GTPase Rab family protein‐facilitated vesicle trafficking (Hurley 2010; Zerial and McBride 2001; Martinez‐Arroyo et al. 2021). Manipulation of these pathways in cancer cells can significantly impair sEV release and cargo sorting capacity (Colombo et al. 2013; Ostrowski et al. 2010; Gorji‐bahri et al. 2021). It has been observed that the absence of Annexin A1 (ANXA1), a key promoter and regulator of PaC development and drug resistance (Bai 2004; Chen et al. 2012; Komoto et al. 2010), dramatically decreases the quantity of secreted sEVs by PaC cells (Pessolano et al. 2018). Among the key components of the ESCRT machinery, ALG‐2‐interacting protein X (ALIX) and tumour susceptibility gene 101 (TSG101) play major roles in sEV biogenesis. ALIX functions as an ESCRT‐associated protein that facilitates membrane budding and cargo selection (IAVELLO et al. 2016), while TSG101 is a core component of ESCRT‐I that mediates the sorting of ubiquitinated proteins into intraluminal vesicles (ILV) (Hadizadeh et al. 2022). Notably, ALIX and TSG101 have been shown to interact directly or within a shared complex, coordinating the formation of ILV within multivesicular bodies (MVBs) (Larios et al. 2020). This interaction underscores their cooperative function in regulating ESCRT‐dependent sEV pathways and supports their classification as hallmark markers and effectors of sEV production. Overall, inhibition of sEV biogenesis can potentially disrupt communication between PaC and its TME, thus hindering tumour progression and metastasis.

Recent studies have identified that the metastasis suppressor N‐myc downstream‐regulated gene 1 (NDRG1) can potently inhibit the cross‐talk between PaC cells and stromal PSCs (Geleta et al. 2021, 2022). Intriguingly, NDRG1 has also been suggested to influence endosomal sorting and EV biogenesis (Pietiäinen et al. 2013; Kachhap et al. 2007; Ortega et al. 2019), although its effects on PaC EV secretion and EV cargo packaging have never been assessed. NDRG1 is commonly expressed in the cytoplasm and nuclei of epithelial tissues and was identified as a metastasis suppressor (Kovacevic and Richardson 2006). In normal cells, NDRG1 regulates multiple cellular pathways and processes relating to differentiation (van Belzen et al. 1997; Chen et al. 2009), lipid synthesis (Hunter et al. 2005), and apoptosis (Stein et al. 2004). Accumulating evidence suggests that NDRG1 plays an important inhibitory role in the progression and metastasis of PaC (Sahni et al. 2020; Kim‐Fuchs et al. 2014; Kovacevic et al. 2011; Angst et al. 2010). This is underscored by studies showing how NDRG1 inhibits tumour growth (Ellen et al. 2008), migration and metastasis (Sun et al. 2013), angiogenesis (Fang et al. 2014) and a plethora of oncogenic signalling pathways in PaC cells (Geleta et al. 2021, 2022; Menezes et al. 2019; Kovacevic et al. 2013). Expression of NDRG1 in cancer cells can also influence the TME (Sevinsky et al. 2018; Li et al. 2021). In PaC cell models, Geleta et al. demonstrated that upregulation of NDRG1 can suppress the formation of desmoplasia by disrupting the communication between PSCs and PaC cells. This study also found that thiosemicarbazone iron‐binding agents, which potently up‐regulate NDRG1, effectively inhibited PaC growth and metastasis in vivo (Geleta et al. 2021, 2022). Notably, NDRG1 expression is upregulated by diverse stimuli, most prominently iron depletion, hypoxia/HIF signalling, and cellular stress pathways (Lane et al. 2013; Le and Richardson 2004). Further, epigenetic regulators such as KDM1A inhibitors and 5‐Aza‐2'‐deoxycytidine also robustly induce NDRG1 by relieving its transcriptional repression (Angst et al. 2010; Mircetic et al. 2023; Han et al. 2013). These studies highlight some of the potential pharmacological approaches to manipulate NDRG1 expression in cancers. However, the mechanisms by which NDRG1 influences the TME and particularly its effects on PaC‐PSC communication remain to be elucidated.

In the current study, we demonstrate for the first time that NDRG1 functions as a regulator of sEV biogenesis in PaC. Our findings reveal that NDRG1 significantly reduces sEV secretion by directly interacting with and promoting the proteasomal degradation of ALIX, a key component of the ESCRT machinery. This interaction substantially alters sEV cargo composition, particularly depleting tumour‐promoting factors such as TGF‐β1. Furthermore, NDRG1 overexpression attenuates PSC activation by sEVs, reduces CAF marker expression, and limits desmoplastic reactions. Additionally, we show that NDRG1 also reduces cancer cell uptake of TME‐derived sEVs while promoting their lysosomal degradation. Together, these findings establish a novel mechanism by which NDRG1 inhibits tumour‐stroma cross‐talk in PaC and reveals NDRG1 as a potential therapeutic target to modulate sEV‐mediated influence on the TME.

## 2 Materials and Methods

### 2.1 Cell Culture

The human pancreatic cancer cell lines PANC‐1 and MIAPaCa‐2 were purchased from the American Type Culture Collection (Rockville, MD). Cells were authenticated based on viability, recovery, growth, morphology, and cytogenetic analysis by the provider. PANC‐1 and MIAPaCa‐2 cells were both derived from the epithelial cells of pancreatic carcinomas. Pancreatic stellate cells (CAT#: 3830), which are derived from a single healthy donor, were purchased from ScienCell Research Laboratories (Carlsbad, CA). PANC‐1 and MIAPaCa‐2 cells were grown in Dulbecco's modified Eagle's medium (DMEM) (Gibco, CAT#:11965092). They were supplemented with 10% (v/v) fetal bovine serum (FBS), 1% (v/v) nonessential amino acids, 1% (v/v) sodium pyruvate, 1% penicillin/streptomycin (Invitrogen). In addition, MIAPaCa‐2 cells were also supplemented with 2.5% horse serum (Gibco). PSCs were cultured with Iscove's Modified Dulbecco's Medium (IMDM), supplemented with 10% FBS and 1% penicillin/streptomycin. Culture media were tested for mycoplasma every 3 months. All cells were grown in a tissue culture incubator at 37°C with 5% CO_2_ and sub‐cultured by standard methods.

### 2.2 Transfection

Both PANC‐1 and MIAPaCa‐2 cells were transfected with pcDNA3.1MycHisA(−) plasmids containing wild type NDRG1 (NDRG1), which were kindly provided by Prof. Yoel Sadovsky (University of Pittsburgh, Pennsylvania; [Shi et al. 2013]). Empty pcDNA3.1MycHisA(−) plasmids were used as vector controls (VC; Invitrogen). To silence NDRG1, MIAPaCa‐2 cells were transfected with an NDRG1 shRNA Plasmid (NDRG1*^KD^*) or a shRNA negative control plasmid (NC; Abbexa, USA). All stably transfected cells were maintained in the presence of G418 (300 µg/mL) as described previously (Chen et al. 2012, Kovacevic et al. 2013).

### 2.3 EV Isolation

**FIGURE 1:**
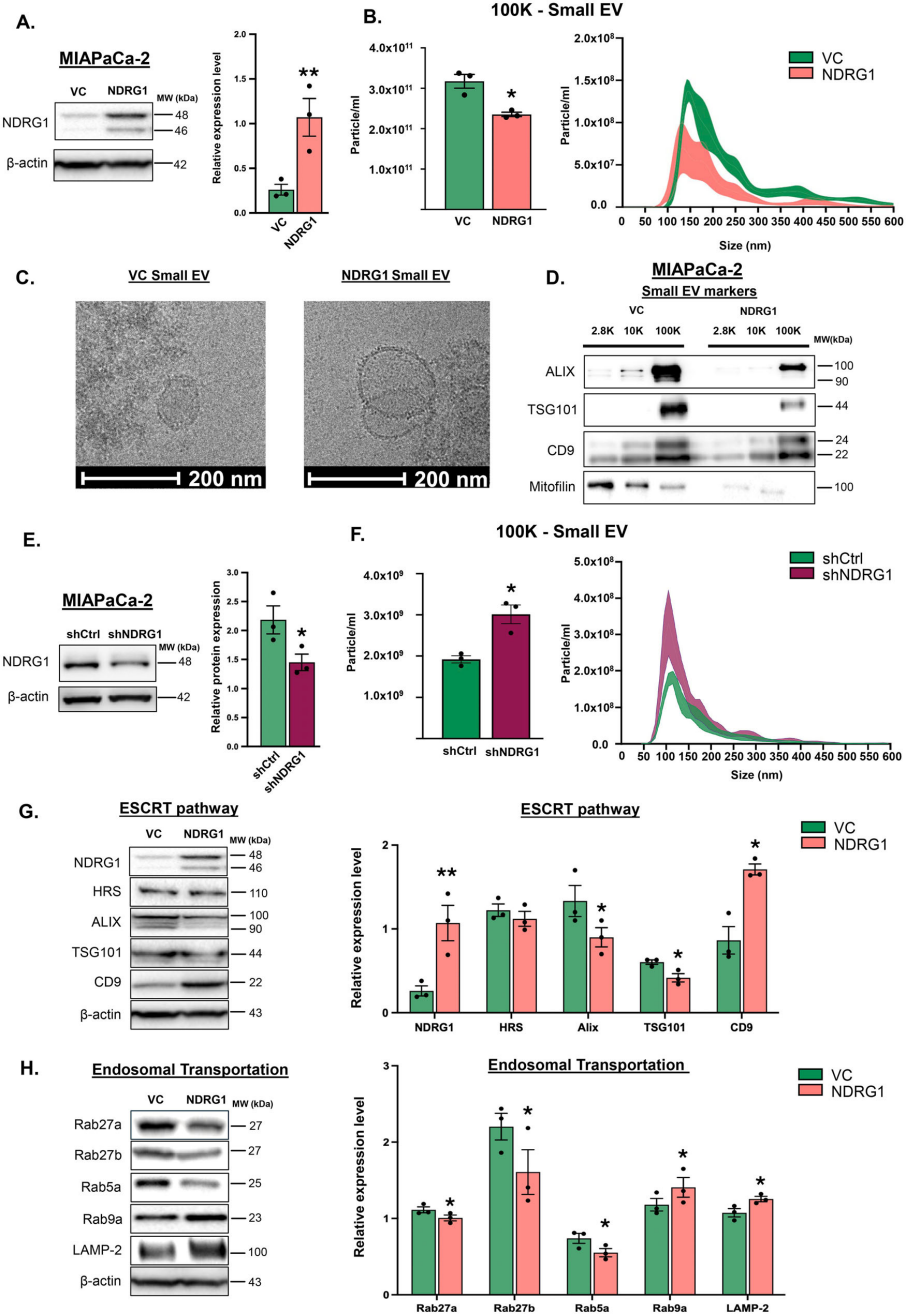
NDRG1 reduces extracellular vesicle (EV) production and biogenesis pathways in pancreatic cancer cells. (A) Representative Western blot images showing NDRG1 expression in MIAPaCa‐2 vector control (VC) cells and MIAPaCa‐2 cells stably transfected to over‐express NDRG1 (NDRG1), with β‐actin as a loading control. The accompanying densitometry analysis quantifies relative NDRG1 expression, with each dot representing an independent experiment (*n* = 3). (B) NTA showing the concentration of sEV isolated from VC or NDRG1 cells with each dot representing an independent experiment. The size distribution graph depicts the particle size profile of sEV measured by NTA, with shaded regions representing the standard error of the mean (SEM), calculated from three independent experiments (*n* = 3). (C) Representative transmission electron microscopy (TEM) images of sEVs isolated from MIAPaCa‐2 VC and NDRG1 cells. Scale bar: 200 nm. (D) Representative Western blot images of sEV markers (ALIX, TSG101, and CD9) and mitochondrial marker (Mitofilin) in 2.8K, 10K, and 100K EV fractions isolated from MIAPaCa‐2 VC and NDRG1 cells. (E) Representative Western blot images showing NDRG1 expression in MIAPaCa‐2 silence control (shCtrl) cells and MIAPaCa‐2 cells stably transfected to knock‐down NDRG1 (shNDRG1), with β‐actin as a loading control. The accompanying densitometry analysis quantifies relative NDRG1 expression, with each dot representing an independent experiment (*n* = 3). (F) Nanoparticle tracking analysis (NTA) showing the concentration of sEVs isolated from shCtrl or shNDRG1 cells with each dot representing an independent experiment (*n* = 3). The size distribution graph depicts the particle size profile of sEV measured by NTA, with shaded regions representing the standard error of the mean (SEM), calculated from three independent experiments. (G) ESCRT pathway proteins (HRS, ALIX, TSG101, and CD9) and (H) endosomal trafficking regulators (Rab27a, Rab27b, Rab5a, Rab9a, and LAMP‐2) in VC or NDRG1 MIAPaCa‐2 cells. The bar graphs adjacent to the blots represent the densitometric analysis, which was normalized to β‐actin. Each dot represents an independent experiment (*n* = 3), and bars indicate the mean ± SEM. Statistical significance was determined using Student's *t*‐test and is indicated as *p < 0.05; **p < 0.01.

To isolate EVs, PaC or PSC cells were first grown in their normal complete media for 48 h until 75% confluency, washed with warm PBS, followed by 48 h culture in FBS‐free media, after which the overlaying media was collected. This conditioned media (CM) then underwent 3 × 300 RCF (5 min) centrifugation steps at 4°C to remove cell debris, followed by 1 × 2800 RCF (10 min) centrifugation at 4°C to isolate large oncosomes and apoptotic bodies. The supernatant then underwent gradient ultracentrifugation at 10,000 RCF at 4°C for 45 min to isolate 10K EVs (Large EVs). Small EVs were then collected by ultracentrifuging the remaining supernatant at 100,000 RCF at 4°C for 65 min (Figure 1A). EV pellets were suspended in 200 µL of sEVs‐free PBS or 100 uL of RIPA buffer (for protein extraction) and stored at −80°C until further use. These methods for enriching different EV populations are well established (Haqqani et al. 2013; Minciacchi et al. 2015; Morello et al. 2013; Hosseini‐Beheshti et al. 2012; Ahmadzada et al. 2023). Protein was extracted from remaining cancer cells using protein lysis buffer for Western blotting or Pierce IP Lysis Buffer (ThermoFisher Scientific) for co‐immunoprecipitation.

### 2.4 NanoSight Particle Tracking Analysis and ZetaSizer Ultra Sizing

Isolated sEVs were analysed using the NanoSight NS300 nanoparticle characterization system (NTA; Nanosight Ltd., UK) for their size and concentration. EV samples were diluted in particle free PBS to a measurable concentration between 0.5 × 10^8^ and 5 × 10^9^ particles/ml. During the analysis, 1 mL of EV samples were injected into a viewing unit using a controlled syringe system. In the viewing unit, a monochromatic laser beam (488 mm) was applied to the diluted samples. Five independent recordings were taken per sample for concentration and size distribution analysis. MIAPaCA‐2 VC and NDRG1 derived EVs with same dilution factors were also examined with Malvern ZetaSizer Ultra (ATA Scientific Pty Ltd, AU) for sizing of EVs.

### 2.5 SiRNA Reconstitution and Reverse Transfection

Lyophilized ALIX siRNAs (Product IDs: s19465 (siALIX #1); s19466 (siALIX #2); ThermoFisher Scientific) and Silencer Select Negative Control #1 (siCtrl; Cat: #4390843; ThermoFisher Scientific) were reconstituted to a stock concentration of 100 µM in nuclease‐free water, aliquoted, and stored at −80°C. Immediately prior to transfection, working solutions were diluted to 10 µM. MIAPaCa‐2 cells were reverse‐transfected utilizing Lipofectamine RNAiMAX (ThermoFisher Scientific). Briefly, transfection complexes were formed by incubating the respective siRNAs and Lipofectamine RNAiMAX reagent in Opti‐MEM Reduced Serum Medium (ThermoFisher Scientific) for 20 min at room temperature. Complexes were dispensed into 6‐well plates, and cells were subsequently seeded at a density of 3 × 10^5^ cells per well in 2.5 mL of antibiotic‐free growth medium. The final assay volume yielded an effective siRNA concentration of 10 nM. A mock transfection containing only the Lipofectamine RNAiMAX reagent (Lipo only) was included as a vehicle control. All standard‐scale experimental conditions were performed in triplicate and incubated under standard conditions, with ALIX knock‐down confirmed by Western blot.

### 2.6 SiALIX Conditioned Media Generation for Exosome Isolation

To generate sufficient starting material for downstream sEV isolation from cells transiently transfected with Lipo only, siCtrl and siALIX, the reverse transfection protocol was volumetrically scaled to a 20 mL final culture volume per condition. At 48 h post‐transfection, the complete growth medium was aspirated to remove exogenous serum‐derived EVs. Cells were washed with sterile PBS and subsequently cultured in medium supplemented with exosome‐free FBS (Cat#: EXO‐FBS‐250A‐1, System Biosciences). After an additional 48 h incubation period, the exosome‐enriched conditioned medium was harvested and cleared of cellular debris. Small EV isolation and subsequent NTA were performed as described in Sections 2.3 and 2.4, respectively.

### 2.7 Small EV Cryo‐EM Data Acquisition

EV samples (4.5 µL) were applied to glow discharged Quantifoil R2/2 copper grids (Quantifoil Micro Tools) and blotted for 2.5 s in a 95% humidity chamber, then plunged in liquid ethane using a Leica EM GP device (Leica Microsystem). The grids were imaged using a Talos Arctica cryoTEM (Thermo Fisher Scientific) and operated at 200 kV, with the specimen maintained at liquid nitrogen temperatures. Images were recorded on a Falcon 3EC direct detector camera operated in linear mode.

### 2.8 Protein Extraction

PaC cells were cultured in FBS‐free media for 48 h prior to protein extraction. Protein extraction was performed as described previously (Kovacevic et al. 2008), using freshly‐made lysis buffer (10 mM Tris buffer, 150 mM NaCl, 5% SDS, 1% Triton X‐100, 1 mM EDTA, 0.04 mM NaF, 4% protease inhibitor (Roche, Germany) and 2x PhoSTOP (Roche, Germany)). Extracted lysates were sonicated on ice, followed by centrifugation at 4°C, 14,000 RPM for 40 min. The supernatant was stored at −80°C. Protein concentration was measured using the BCA protein assay (ThermoFisher Scientific). EV protein concentration was measured using a microBCA kit (ThermoFisher Scientific). Protein absorbance was measured at 562 nm.

### 2.9 Western Blotting

Western analysis was performed via standard procedures (Kovacevic et al. 2013) using antibodies listed in Table 1. Western blots were imaged using Immobilon ECL Ultra Western HRP Substrate (MERCK, CAT#: WBULS0100) with the Bio‐Rad ChemiDoc MP Imaging System (Bio‐Rad. Australia). Band intensity was analysed with the Bio‐Rad Image Lab software.

**TABLE 1:** Antibodies used for immunoblotting and immunofluorescence. Primary and secondary antibodies used for Western blotting (WB) and immunofluorescence (IF), including the working dilutions for each application. All antibodies were used according to the manufacturer's instructions, with dilutions optimized for analysis.

Antibody	Catalogue #	Company	Dilution (WB)	Dilution (IF)
NDRG1	9485S	Cell signalling technology	1:1000	1:200
NDRG1	J6271‐2D7	Sigma–Aldrich		1:200
ALIX	92280	Cell signalling technology	1:1000	1:800
Ubiquitin	E4I2J	Cell signalling technology	1:1000	
RAB5A	46449	Cell signalling technology	1:1000	1:200
RAB27A	69296	Cell signalling technology	1:1000	1:200
RAB27B	17572	Cell signalling technology	1:1000	1:500
RAB9A	95394	Cell signalling technology	1:1000	1:200
HRS	A300‐989A‐M	Bethyl Laboratories	1:1000	
TSG101	ab30871	Abcam	1:1000	
CD9	13403	Cell signalling technology	1:1000	
LAMP2	ab25631	Abcam	1:500	1:500
TGF‐b1	ab215715	Abcam	1:1000	
aSMA	19245	Cell signalling technology	1:1000	
FAP	66562	Cell signalling technology	1:1000	
COL1A1	72026	Cell signalling technology	1:1000	1:1000
p‐ERK1/2 (Thr202/Tyr204)	9101	Cell signalling technology	1:500	
p‐P38a (Thr180/Tyr182)	9211	Cell signalling technology	1:500	
Total ERK1/2	4695	Cell signalling technology	1:1000	
Total P38a	8690	Cell signalling technology	1:1000	
β—actin	BA3R	Thermo Scientific	1:10000	
Anti‐rabbit IgG, HRP‐linked Antibody	7074	Cell signalling technology	1:10000	
Goat anti‐Mouse IgG (H+L) Secondary	31430	Thermo Scientific	1:10000	
Anti‐rabbit IgG (H+L), F(ab')2 Fragment (Alexa Fluor 594 Conjugate)	8889	Cell signalling technology		1:500
Anti‐mouse IgG (H+L), F(ab')2 Fragment (Alexa Fluor 488 Conjugate)	4408	Cell signalling technology		1:500

### 2.10 Immunofluorescent Staining and Microscopy

Transfected MIAPaCa‐2 cells were seeded into 24‐well plates onto sterile 1 cm round coverslips. Cells were incubated in their standard media for 24 h, followed by a further 48 h incubation in FBS‐free media. Cells were fixed with 4% paraformaldehyde (PFA) in PBS for 10 min, washed and permeabilized with 0.1% Triton X‐100/PBS for 15 min at room temperature. After blocking with 5% BSA/PBS (45 min), the cells were incubated with the primary antibody diluted in 1% BSA/PBS overnight at 4°C. Antibodies used and their concentration can be found in Table 1. After washing, cells were incubated with secondary antibodies at a dilution of 1:500 in 1% BSA/PBS for 1 h, washed with 0.2% BSA/PBS (3 × 10 min), stained with DAPI for 3 min (NucBlue Fixed Cell ReadyProbes Reagent (DAPI), CAT#: R37606, Invitrogen) and mounted onto glass slides using mounting medium (ProLong Diamond Antifade Mountant, CAT#P36961, Invitrogen) and allowed to cure at room temperature for 24 h prior to imaging. Images were acquired with the Leica Stellaris 8 confocal imaging system.

### 2.11 Proximity Ligation Assay

VC and NDRG1‐overexpressing MIAPaCa‐2 cells were seeded on to 8‐well chamber slides (CAT#: 154534, ThermoFisher Scientific). Once cells reached 75% confluency, they were cultured in FBS free media for 48 h. Cells were fixed with 4% PFA at room temperature for 10 min, then they were washed and permeabilized with 0.1% Trion‐X‐100/PBS for 15 min. Proximity ligation assay (PLA) was performed using the Duolink In Situ Red Starter Kit Mouse/Rabbit (CAT#: DUO92101, Sigma–Aldrich) following the manufacturer's protocol using ALIX (CAT#: 92280, Cell Signalling technology, 1:800 dilution) and NDRG1 (CAT#: J6271‐2D7, Sigma–Aldrich, 1:200 dilution) antibodies. Images were taken on Leica Stellaris 8 confocal imaging system with 20x magnification.

### 2.12 Co‐immunoprecipitation and MG132 Protein Degradation Assessment

Protein lysates were extracted from MIAPaCa‐2 VC and NDRG1‐overexpressing cells using Pierce IP Lysis Buffer supplemented with 4% protease inhibitor cocktail (Roche, Germany), after serum starvation for 48 h. For immunoprecipitation, NDRG1 Rabbit monoclonal antibody (CAT #: 9485S, Cell Signalling Technology, 1:50 dilution), ALIX Rabbit monoclonal antibody (CAT #: 92880S, Cell Signalling Technology, 1:50 dilution), or Rabbit IgG isotype control (Cell Signalling Technology, CAT#: 3900, concentration matched to antibody) were incubated with Dynabeads (ThermoFisher Scientific) for 4 h at 4°C on a rotating mixer. Subsequently, 30 µL of bead‐antibody complexes were incubated with 200 µL of protein lysate (2 µg/µL) overnight at 4°C with rotation. Prior to Western blotting, samples were thoroughly washed with IP Lysis Buffer. Input controls were taken before bead‐antibody incubation, and supernatant controls (15 µL, 2 µg/µL) were collected post‐incubation. To assess ALIX ubiquitination, cells were treated with 2.5 µM MG132 (CAT#: 2194, Cell Signalling Technology) for 24 h prior to extraction and Co‐IP. Western blotting analysis employed ALIX antibody (CAT#: 92880S, Cell Signalling Technology, diluted 1:1000), NDRG1 antibody (Abcam, Cat#: ab37897, diluted 1:1000), and ubiquitin antibody (CAT#: E4I2J, Cell Signalling Technology, diluted 1:1000), following the method described in Section 2.10.

### 2.13 Cycloheximide Chase Protein Half‐life Assay

MIAPaCa‐2 VC and NDRG1 cells were treated with cycloheximide (CHX, 15 µg/mL; CAT#: 2112, Cell Signalling) diluted in serum free media to inhibit protein synthesis. CHX treatment was administered for 0, 4, 8, 16, 24, 36, and 48 h, followed by protein extraction and Western blotting.

### 2.14 NDRG1 And ALIX Protein Remodelling and Docking

The 3D models of NDRG1, ALIX and their predicted interaction were generated using AlphaFOLD3 (Abramson et al. 2024). Amino sequence data were obtained from publicly available UniProt. The lowest energy state model was chosen for optimal representation. Docked profile and polar interaction were then visualized using PyMOL.

### 2.15 Label‐Free Qualitative Proteomic and DIA‐NN Library‐Free Peptide Search

SEVs and 10K EV samples derived from MIAPaCa‐2 VC and NDRG1 cells were suspended in 1x RIPA buffer (Abcam) with 12.5% protease and phosphatase inhibitors (Roche, Germany). Quantitative proteomic analysis of cancer cell derived sEVs was performed by Sydney Mass Spectrometry using standard procedures (Jalaludin et al. 2023). SEVs samples were run on DIA mode on HFX4 for peptide search. The HFX4 output files were uploaded to DIANN version 1.8.1 and processed in a library free search against Human genome for peptide identification.

### 2.16 EV Proteomic Data Analysis

Raw proteomic data generated via DIA‐NN were filtered to include only proteins identified in all three independent replicates across both VC and NDRG1‐derived sEV groups. Protein abundance levels were log​10‐transformed to normalize the distribution. Differentially expressed proteins (DEPs) were defined using a significance threshold of *p* < 0.01 (corresponding to −log10​(*p*) > 2). No minimum fold‐change magnitude threshold was applied for the volcano plot visualization (i.e. ∣log2​Fold Change∣>0). For subsequent heatmap analysis, a broader significance threshold of *p* < 0.05 was utilized, with proteins ranked by their absolute fold change. Finally, t‐distributed Stochastic Neighbor Embedding (t‐SNE) was performed in R to visualize sample clustering and proteomic profile similarity between groups.

### 2.17 Phospho‐Kinase Array Assay

PSCs seeded in 10 cm culture plates were incubated with MIAPaCa‐2 VC and NDRG1‐derived sEVs (2 × 10^10^ particles) for 1 h in Serum Free media, followed by protein extraction of the PSC lysate. The phosphorylation status of 37 human kinases in the PSC lysates were assessed with the proteome profiler human phospho‐kinase array kit (R&D System, CAT#: ARY003C), with 600 µg of PSC lysate being applied to the membrane. membranes were imaged with the Bio‐Rad ChemiDoc MP imaging system.

### 2.18 PSC Functional Assay

For functional assays, PSCs were treated with sEVs freshly isolated from MIAPaCa‐2 VC and NDRG1‐overexpressing cells at 90% confluency. Following isolation, sEVs were resuspended in serum‐free IMDM. Initial NTA analysis across three independent experiments demonstrated that VC cells secreted approximately 25% more sEVs than NDRG1‐overexpressing cells (Figure 1B). To account for this, VC‐derived sEV input was reduced by 20% prior to downstream functional assays. Subsequent NTA analysis performed following this adjustment (using an independent instrument) indicated that particle concentrations between VC and NDRG1 sEV preparations were more closely aligned, with a residual difference of approximately 14% (Figure S1A). For MAPK activation analysis, PSCs were treated with sEVs for 0, 15, 30, 45, and 60 min in serum‐free media to eliminate FBS contaminants. To assess PSC activation and collagen production, PSC cells were seeded on a 96‐well plate and incubated with cancer cell derived sEVs for 24 h in serum‐free media. For the sEV‐mediated TGF‐β neutralization assay, PSCs were cultured in 6‐well plates until they reached 90% confluence, followed by washing with warm, sterile PBS. The PSCs were then incubated in 1.5 mL of serum‐free IMDM media containing normalized amounts of: (i) VC‐derived sEVs; (ii) NDRG1‐derived sEVs; (iii) VC‐derived sEVs combined with 1 µg/mL of TGF‐β neutralizing antibody (α‐TGF‐β; Cat#: MAB1835, R&D Systems), or; (iv) NDRG1‐derived sEVs combined with 1 µg/mL of α‐TGF‐β for 24 h. Prior to adding the sEV‐containing media to PSCs, all 4 sEV preparations were first incubated at 37°C for 2 h to facilitate α‐TGF‐β binding to its target before cell exposure. Following sEVs treatment, cell lysates were collected for protein quantification and immunoblotting. Additionally, PSCs were seeded in 96‐well glass plates, incubated with cancer cell derived PKH67 stained sEVs for 24 h, following by the immunofluorescence protocol (described in Section 2.10). Fixed PSCs were examined for sEV uptake and collagen production with immunofluorescent microscopy. Images were taken with a Leica Stellaris 8 confocal microscope.

### 2.19 Small EV Uptake Assay

After isolation from PSCs, MIAPaCa‐2 and PANC‐1 parental cells, sEVs were concentrated and suspended in 200 µL of PBS. SEVs were labelled with a PKH67 Green Fluorescent Cell Linker Kit (CAT#: PKH67GL‐1KT, Sigma–Aldrich) following the manufacturer's protocol. Labelled PSC‐derived sEVs were then resuspended in serum free media and added to either MIAPaCa‐2 VC or MIAPaCa‐2 NDRG1 cells for 24 h. Additional validation was done using a protein‐based labelling approach, with the Alexa Fluor 488 NHS Ester (Succinimidyl Ester; CAT#: A20000 ThermoFisher Scientific) used to label sEVs (following the manufacturer's protocol) prior to incubation with MIAPaCa‐2 cells. Prior to imaging, these cells were fixed with 4% PFA and washed with PBS to remove residual sEVs. Following DAPI staining and mounting, immunofluorescent imaging was performed to detect the labelled PSC‐derived sEVs using a Leica Stellaris 8 confocal microscope with a 488 nm laser.

### 2.20 Small EV‐Lysosome Colocalization With Live Cell Confocal Microscopy

MIAPaCa‐2 parental cell‐derived sEVs were collected and stained with PKH67 dye as described above. MIAPaCa‐2 VC and NDRG1 cells were seeded into black polystyrene microplates (CAT#: CLS3603, Sigma–Aldrich) cultured under standard conditions. Upon reaching 75% confluency, they were treated with LysoTracker Deep Red (5 nM) (CAT#: L1249, Themo Fisher Scientific) and Hoechst live nuclei stain (2 µg/mL) for 1 h at 37°C. FBS‐free media containing PKH labelled sEVs (approx. 1 × 10^9^) was then added to either VC or NDRG1 cells, followed by a 2.5 h incubation. Confocal live‐cell imaging was performed using a Leica Stellaris 8 microscope for 16 h at 37°C and 5% CO_2_, capturing 16 ROIs/sample every 15 min (total 65 time points). Data analysis was performed using a custom Java script to calculate the overlay score of lysosome and sEV fluorescent signals. Fluorescent signals were normalized to nuclear DAPI intensity.

### 2.21 Data Analysis

Densitometric data from Western blot analysis were evaluated using Bio‐Rad Image Lab software. Protein expression levels were quantified as adjusted volumes and normalized to β‐actin. Immunofluorescence intensities were measured in LAS X software, and colocalization scores (Pearson's correlation coefficients) were calculated using the Fiji Coloc2 plugin. For the sEV–lysosome colocalization experiment, a custom JavaScript using Fuji was employed to batch‐calculate the overlay scores between lysosomal and sEV fluorescent signals. All graphs were generated in GraphPad Prism, and statistical significance was determined using Student's *t*‐test. Experiments were performed using independently cultured cells and independently prepared EV isolations on different days. Unless otherwise indicated, data represent at least three independent replicates.

## 3 Results

### 3.1 NDRG1 Reduces sEV Release by Interfering With Intracellular Trafficking and the ESCRT Pathway

NDRG1 was found to inhibit cross‐talk between PaC cells and PSCs, disrupting key oncogenic signalling pathways, although the precise mechanisms underlying this inhibitory effect remain to be fully elucidated (Geleta et al. 2021, 2022; Chang et al. 2023). To investigate whether NDRG1 can influence sEV biogenesis in PaC, we generated a stable NDRG1‐overexpressing MIAPaCa‐2 cell line and compared it with a vector control (VC) MIAPaCa‐2 line that exhibits low NDRG1 expression. Immunoblot analysis confirmed successful overexpression, with NDRG1 levels approximately 5‐fold higher in the overexpressing cells compared to VC cells (Figure 1A).

To isolate and characterize EVs derived from both NDRG1‐overexpressing and VC MIAPaCa‐2 cells, we used the well‐established differential ultracentrifugation protocol to enrich for either: (i) large oncosomes following centrifugation at 2800 RCF (2.8K EVs); (ii) large EVs (microvesicles) following centrifugation at 10,000 RCF (10K EVs), and (iii) sEVs following centrifugation at 100,000 RCF (100K EVs) (Brennan et al. 2020). Nanoparticle tracking analysis revealed that NDRG1‐overexpressing MIAPaCa‐2 cells secreted significantly fewer sEVs than VC cells (Figure 1B). Quantitative analysis showed an approximately 25% reduction in particle number in the NDRG1‐overexpressing condition (2.4 × 10^11^ vs. 3.2 × 10^11^ particles/ml). The size distribution profiles further confirmed this reduction across the characteristic sEV size range (30–200 nm), with NDRG1‐overexpressing cells showing a lower peak and narrower distribution (Figure 1B). In addition, to further validate our EV isolation protocol, we also examined the size profile of the three subtypes of EVs using ZetaSizer Ultra (Malvern Panalytical, UK; Table 2). Each EV population was observed to have distinct particle sizes, with 2.8K EVs having an average size of 1240–1600 nm, 10K EVs being predominantly in the 500–600 nm range, and sEV being approximately 200 nm.

**TABLE 2:** UltraSizer report. Z‐average size distribution and related statistical parameters of extracellular vesicles (EVs) isolated from MIAPaCa‐2 cells, analysed using the UltraSizer system. Measurements were performed on three EV fractions (2.8K, 10K, and 100K) from both vector control (VC) and NDRG1‐overexpressing cells. For each measurement, 10 µL of EV suspension was further diluted in 1 mL of ultrapure water. The table reports the mean particle size (Z‐average, in nm), standard deviation, relative standard deviation (RSD), and the minimum and maximum particle sizes detected.

Z‐average (nm)	Mean	Standard deviation	RSD	Minimum	Maximum
MIAPaCa‐2 VC 2.8K	572.4	166.6	29.11	428.5	754.9
MIAPaCa‐2 VC 10K	575.6	98.83	17.17	497	686.5
MIAPaCa‐2 VC 100k	203.4	9.332	4.589	195.6	213.7
MIAPaCa‐2 NDRG1 2.8K	1615	614.5	38.05	905.6	1981
MIAPaCa‐2 NDRG1 10K	572.4	166.6	29.11	428.5	754.9
MIAPaCa‐2 NDRG1 100K	232.3	2.976	1.281	229.5	235.4

Transmission electron microscopy (TEM) of the MIAPaCa‐2 VC or NDRG1 derived sEV fractions confirmed the morphological characteristics of isolated sEVs, revealing the typical double‐layered membrane structures with a diameter less than 200 nm from both conditions (Figure 1C). Notably, the ultrastructural features appeared consistent between VC and NDRG1 sEVs, suggesting that NDRG1 affects quantity rather than basic morphology of sEVs.

To further characterize the EV fractions, immunoblotting of each fraction was performed, confirming the presence of the sEV surface marker CD9 and endosomal markers TSG101 and ALIX, which were predominantly enriched in the 100K sEV fraction (Figure 1D). As this analysis was intended to assess the presence and enrichment of EV markers across fractions rather than to quantitatively compare protein expression levels, equal amounts of total protein were loaded for each sample. Together with sEV size profiles and TEM imaging, these findings are consistent with established EV characterization approaches and comply with the minimal information for studies of EVs (MISEV) guidelines (Dilsiz 2024; Saint‐Pol and Culot 2025; Wang et al. 2022). Importantly, the mitochondrial marker, Mitofilin was detected primarily in the 2.8 and 10K fractions and was detected with minimal expression in the sEVs fraction (John et al. 2005), confirming minimal cellular contamination in our sEV preparations. Comparing the VC and NDRG1 fractions, we observed reduced levels of ALIX and TSG101 in the sEV fraction from NDRG1‐overexpressing cells compared to VC cells, suggesting that NDRG1 may reduce sEV production and affect the incorporation of these ESCRT components into sEV.

To validate the effect of NDRG1 on sEV production in another PaC cell line, PANC‐1 cells were also transfected to over‐express NDRG1, which also led to a significant reduction in sEV secretion from PANC‐1 NDRG1 cells compared to their VC counterparts (Figure S1B–E). Conversely, NDRG1 silencing in MIAPaCa‐2 cells had the opposite effect, leading to a pronounced increase in sEV release (Figure 1E,F; Figure S1F). Overall, these results validate the inhibitory effect of NDRG1 on sEV secretion by PaC cells.

To explore the underlying mechanism by which NDRG1 reduces sEV release, we first examined key ESCRT pathway proteins, including hepatocyte growth factor‐regulated tyrosine kinase substrate (HRS), TSG101 and CD9, as well as the ESCRT accessory protein ALIX, which plays a central role in sEV biogenesis. Immunoblot analysis of cellular lysates from MIAPaCa‐2 VC and NDRG1 cells revealed that overexpression of NDRG1 was associated with a significant reduction in ALIX and TSG101, while CD9 accumulated intracellularly and HRS remained unchanged (Figure 1G). A similar regulatory role for NDRG1 was also observed in the PANC‐1 cells, where NDRG1 overexpression also led to decreased expression of ALIX and TSG101, while CD9 expression was upregulated (Figure S2A). Notably, HRS, which initiates endosomal sorting (Bache et al. 2003), was significantly up‐regulated by NDRG1 in the PANC‐1 cells (Figure S2A). Notably, ALIX was detected as 2–3 distinct bands in both MIAPaCa‐2 and PANC‐1 cells, which may arise from post‑translational modifications (Feng et al. 2007, Wei et al. 2021).

To further understand how NDRG1 affects intracellular endosome trafficking and MVB formation, the expression of small GTPases, including Rab27a, Rab27b, Rab5a and Rab9a, which are crucial for MVB maturation and membrane fusion (Ostrowski et al. 2010; Skjeldal et al. 2021), and lysosomal marker LAMP‐2 were also examined. Overexpression of NDRG1 in MIAPaCa‐2 cells led to a significant reduction in Rab27a, Rab27b and Rab5a (Figure 1H). In contrast, Rab9a and LAMP‐2 were upregulated in NDRG1‐overexpressing MIAPaCa‐2 cells (Figure 1H), suggesting enhanced lysosomal activity (Mahanty et al. 2016). Similar results were observed in the PANC‐1 cells, where Rab27A and Rab5a were decreased, while LAMP2 was increased in NDRG1‐overexpressing cells (Figure S2B). However, Rab27b showed increased expression, while Rab9a was significantly reduced in NDRG1‐overexpressing PANC‐1 cells when compared to the VC cells (Figure S2B), which was opposite to the expression of these proteins in MIAPaCa‐2 cells. These results suggest the effect of NDRG1 on sEV biogenesis may involve slightly different mechanisms in the 2 PaC cell lines examined.

Silencing NDRG1 (shNDRG1) in MIAPaCa‐2 cells had no significant effect on the ESCRT proteins, although there was a significant increase in Rab27a expression when compared to the negative control (NC) cells (Figure S2C,D). It is important to note that in these latter experiments, NDRG1 was not completely silenced, and this may contribute to the lack of effect on the other proteins examined.

Overall, these findings indicate that NDRG1 overexpression significantly reduces sEV secretion, potentially by altering key components of the ESCRT pathway and modulating intracellular trafficking machinery in PaC cells. The concurrent downregulation of Rab GTPases (Rab27a/b, Rab5a), which promote sEVs secretion, and upregulation of lysosome‐associated proteins (LAMP2, Rab9a), suggests that NDRG1 may redirect MVB content toward lysosomal degradation rather than sEV release. Based on these observations, we next sought to determine whether NDRG1 functionally interacts with specific ESCRT factors to regulate sEV biogenesis.

### 3.2 NDRG1 Interacts With ALIX and Induces Its Proteasomal Degradation

To determine whether NDRG1 functionally associates with the ESCRT pathway, we next examined its relationship with ALIX, a component of the ESCRT machinery that facilitates sEV maturation and cargo loading during MVB sorting (Sun et al. 2015). Given the importance of ALIX in forming ILV, any modulation of ALIX expression or function has the potential to significantly impact sEV‐mediated TME communication (Larios et al. 2020; Monypenny et al. 2018). Indeed, ALIX acts as a critical scaffolding protein that bridges the Syndecan‐Syntenin complex to the ESCRT‐III machinery, a pathway shown to directly regulate the rate of exosome budding and secretion (Roucourt et al. 2015). Studies have demonstrated that the depletion of ALIX can lead to a marked reduction in the release of CD63‐positive sEVs, while its overexpression can enhance the secretion of vesicles with specific protein and miRNA signatures, further influencing downstream signalling in the tumour niche (Iavello et al. 2016; Matsui et al. 2021).

Given this critical role in vesicle biogenesis, we first sought to confirm the dependency of PaC sEV secretion on ALIX in our model. Accordingly, we used two independent siRNAs targeting distinct regions of the ALIX transcript (siALIX #1 and siALIX #2) to transiently knock‐down ALIX protein expression in MIAPaCa‐2 cells (Figure S3A). This targeted depletion resulted in a marked reduction in sEV secretion in response to both ALIX siRNAs when compared to the negative siCtrl and the Lipofectamine‐only control (Figure S3B). This demonstrates that ALIX can directly influence EV secretion in PaC cells.

**FIGURE 2:**
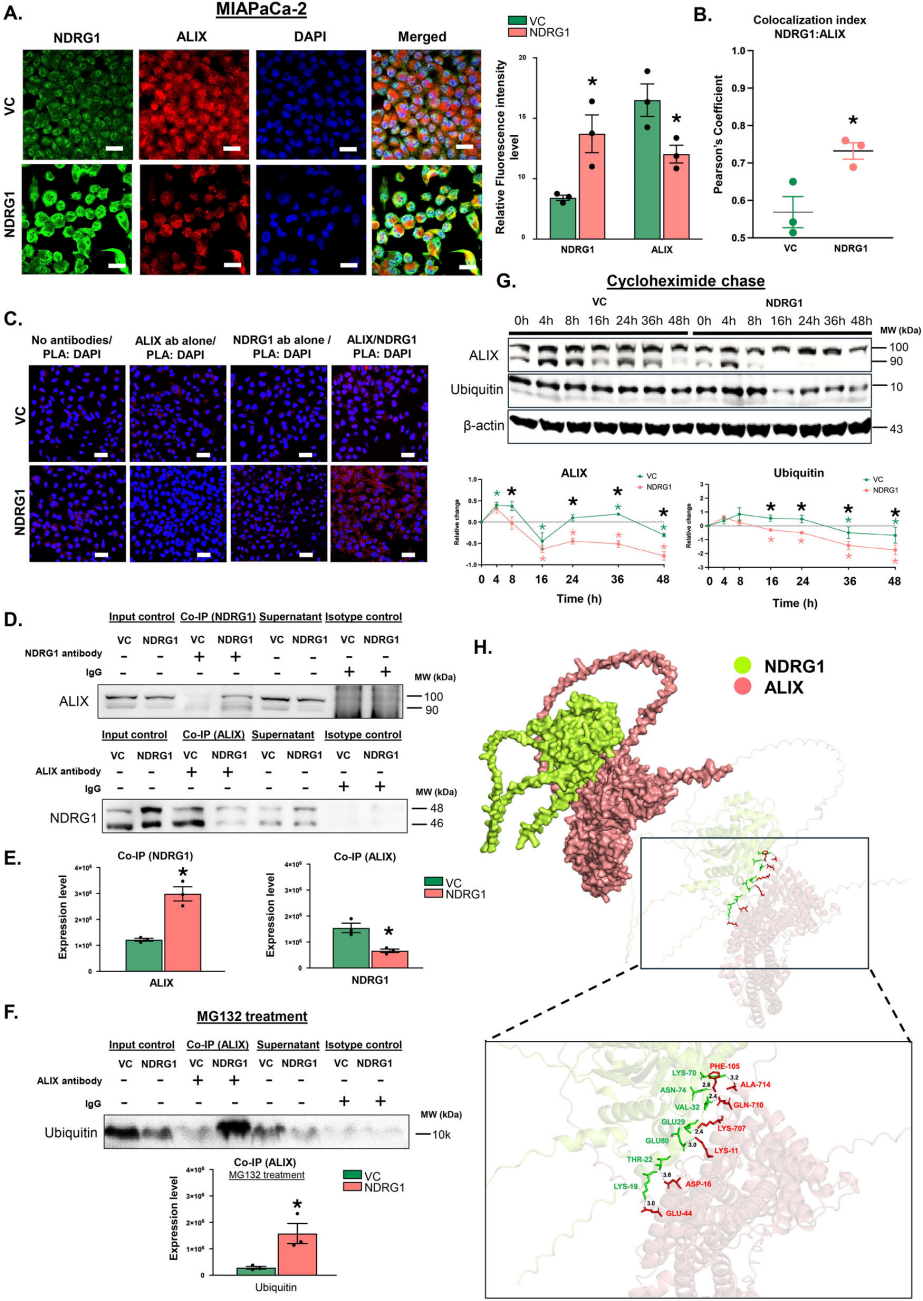
NDRG1 interacts with ALIX and promotes its proteasomal degradation. (A) Representative immunofluorescence images of MIAPaCa‐2 cells expressing vector control (VC) or NDRG1 show staining for NDRG1 (green), ALIX (red), and nuclei (DAPI, blue), with merged images indicating colocalization of NDRG1 and ALIX. Scale bar: 20 µm. Quantification of fluorescence intensity levels are shown in the adjacent bar graph, with each dot representing an independent experiment (*n* = 3) and bars indicating mean ± SEM. (B) Pearson's correlation coefficient analysis quantifies the degree of colocalization between NDRG1 and ALIX. (C) Representative immunofluorescence images of the proximity ligation assay (PLA) in VC and NDRG1 MIAPaCa‐2 cells incubated with no antibody, ALIX antibody (ab) alone, NDRG1 ab alone or NDRG1 and ALIX abs together. Positive PLA staining is shown in red, with DAPI stained nuclei shown in blue. Scale bar: 20 µm. (D) Co‐immunoprecipitation (Co‐IP) experiments confirm the interaction between NDRG1 and ALIX, with ALIX detected in NDRG1 pull‐downs and vice versa. (E) Quantification of Co‐IP data shows levels of ALIX‐NDRG1 interaction, represented as mean ± SEM from three independent experiments. (F) Increased ALIX ubiquitination is observed in NDRG1‐expressing cells following MG132 treatment (2.5 µM, 24 h), and quantification of Co‐IP data shows ALIX ubiquitination, represented as mean ± SEM from three independent experiments. (G) Representative Western blot images demonstrate a cycloheximide (CHX) chase of ALIX and ubiquitin protein levels in VC and NDRG1 cells over a 48 h time‐course following CHX treatment (15 µg/mL), with β‐actin as a loading control. Coloured asterisks (*) indicate significant differences compared to 0 h, while black asterisks indicate significant difference between VC and NDRG1 cells. Quantification of ALIX and ubiquitin levels at each time point was normalized to β‐actin. All Statistical significance was determined using Student's *t*‐test: *p < 0.05. (H) AlphaFold3 protein‐protein interaction prediction of ALIX (pink) and NDRG1 (green). Enlarged view shows the binding amino acid residues from NDRG1 (green) and ALIX (red). Dashed lines indicate polar interactions labelled with distance between binding amino acids (Å3).

Immunofluorescence microscopy performed on MIAPaCa‐2 cells showed that NDRG1 overexpression markedly reduced ALIX levels in the cytoplasm, when compared with VC cells (Figure 2A), aligning with our earlier immunoblot analysis (Figure 1 G,H). Furthermore, Pearson's correlation analysis indicated a close spatial relationship between NDRG1 and ALIX. In NDRG1‐overexpressing cells, the correlation coefficient was substantially elevated (Figure 2B), indicating significant co‐localization at the pixel level. Similar results were observed for Rab5a and Rab9a in MIAPaCa‐2 cells; the significant increase in Pearson's coefficients suggests that the fluorescent signals for these proteins overlap consistently, placing them within the same sub‐cellular compartments. (Figure S3C–F).

Given that ALIX directly interacts with ESCRT‐III and promotes MVB maturation (Sun et al. 2015), this observation raised the possibility that NDRG1 might influence sEV composition by directly altering ALIX's availability and function. To investigate the potential interaction between NDRG1 and ALIX further and assess direct protein–protein interaction, a PLA, which detects proteins residing within close molecular distances (≤40 nm), was used. The PLA signals indicated a notable increase in the NDRG1–ALIX interaction in cells overexpressing NDRG1, with only minimal background signal being observed from control samples where only one antibody (NDRG1 or ALIX) or samples with no antibody were imaged (Figure 2C). This was further supported by co‐immunoprecipitation assays, in which ALIX was detected in the NDRG1 immunoprecipitate and, conversely, NDRG1 was detected in the ALIX immunoprecipitates (Figure 2D), indicating an association between these proteins. As expected, increased levels of ALIX were recovered in NDRG1 immunoprecipitates from NDRG1‐overexpressing cells relative to VC cells (Figure 2E), reflecting the higher abundance of NDRG1 in these samples. Notably, the amount of NDRG1 detected in ALIX immunoprecipitates appeared lower in NDRG1‐overexpressing lysates (Figure 2E), which is consistent with reduced ALIX levels in these cells. Together with the Western blot data (Figure 1G) and immunofluorescence analysis (Figure 2A), these findings are consistent with a model in which ALIX expression and/or stability may be reduced in NDRG1‐overexpressing MIAPaCa‐2 cells.

To assess if the NDRG1‐mediated reduction of ALIX could be linked to lysosomal degradation, we examined the potential co‐localization of ALIX with the lysosomal marker LAMP‐2 in MIAPaCa‐2 VC and NDRG1 cells (Figure S3G). However, we observed no marked increase in co‐localization of these two proteins when comparing VC to NDRG1‐overexpressing cells (Figure S3H). We next investigated the involvement of proteasomal degradation by treating MIAPaCa‐2 VC and NDRG1 cells with the proteasomal inhibitor MG132 for 24 h to inhibit proteasomal turn‐over of ubiquitinated proteins and thus enable detection of protein ubiquitination by Co‐IP. Treatment of cells with MG132 followed by immunoprecipitation of ALIX revealed increased levels of ubiquitin binding to ALIX in the NDRG1‐overexpressing cells (Figure 2F), implicating NDRG1 as a regulator that potentially targets ALIX for proteasomal degradation.

This finding was further substantiated by cycloheximide (CHX) chase experiments, which measure protein turnover by inhibiting new protein synthesis. In VC cells, ALIX levels declined noticeably at 16 h then increased at 24 and 36 h, followed by a reduction at 48 h post‐treatment with CHX. However, in NDRG1‐overexpressing cells, significant ALIX depletion was evident as early as 16 h and remained significantly lower than in VC cells for all subsequent time‐points (Figure 2G). Additionally, the rate of ubiquitin turnover was elevated in the NDRG1‐overexpressing cells, suggesting that increased proteasomal activity accompanies or is driven by the presence of high NDRG1 levels (Figure 2G).

Finally, to gain insight into how NDRG1 might physically interact with ALIX and potentially modulate its function in sEV biogenesis, we performed in silico protein–protein docking using UniProt sequences and AlphaFold3 for molecular interaction (Abramson et al. 2024). These simulations indicated that NDRG1 is binding to multiple regions of the V‐shaped Bro1 domain of ALIX (Figure 2H). The Bro1 domain of ALIX encompasses amino acid 1 to amino acid 359 (Zhai et al. 2011). NDRG1 was shown to bind to ALIX amino acid sequence LYS‐11, ASP‐16, GLU‐44, and PHE‐105. Notably, the Bro1 domain mediates ALIX's interaction with CHMP4B which belongs to ESCRT‐III (Sun et al. 2015; McCullough et al. 2008), and is essential for MVB maturation and cargo loading (Larios et al. 2020; Sun et al. 2015). We also noted that engagement by NDRG1 altered ALIX's conformational state (Figure S4A), which could potentially restrict its access to binding partners (Figure S4B,C). Hence, NDRG1 binding to ALIX may have a dual role, including enhancing the proteasomal degradation of ALIX, while also potentially impairing ALIX‐mediated cargo recruitment and MVB formation. Overall, these data demonstrate that NDRG1 binding to ALIX has significant implications for sEV composition and release in PaC.

### 3.3 NDRG1 Expression Alters EV Protein Cargo

Building on our observation that NDRG1 modulates ALIX in the ESCRT pathway, we performed comprehensive proteomic analysis to determine whether these alterations translate into measurable changes in EV cargo. Using label‐free quantitative mass spectrometry, we compared the protein composition of EVs derived from NDRG1‐overexpressing MIAPaCa‐2 cells with those from VC cells.

**FIGURE 3:**
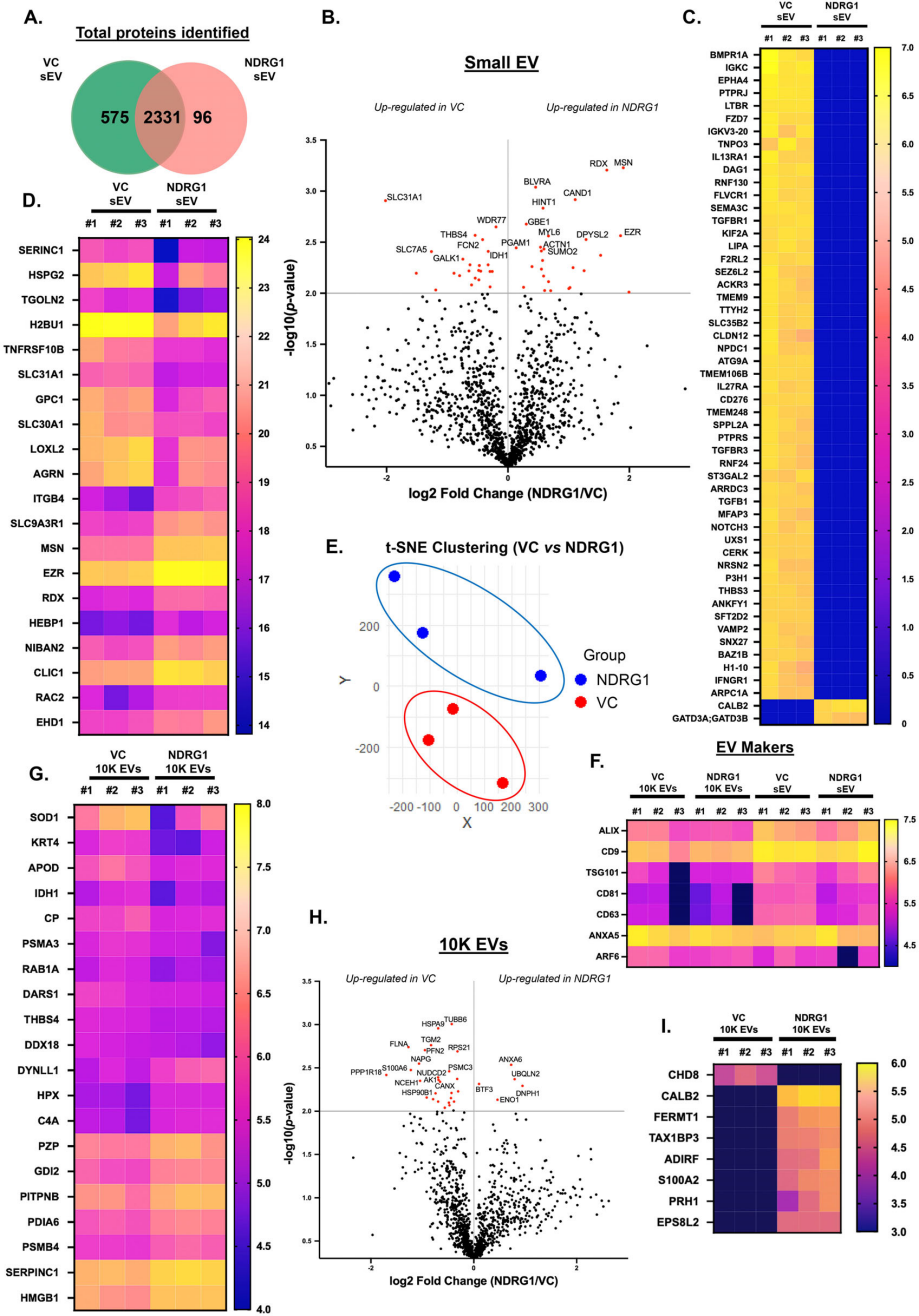
Mass spectrometry proteomic analysis reveals NDRG1 expression affects peptide cargo in EVs. (A) Venn diagram illustrating the number of proteins identified in sEVs from MIAPaCa‐2 VC or NDRG1‐overexpressing cells. (B) Volcano plot showing the log2 fold change (NDRG1/VC) on the x‐axis and ‐log10 (*p* value) on the y‐axis, representing differentially expressed proteins in sEVs isolated from MIAPaCa‐2 cells. Red dots indicate proteins with significant differential expression (*p* < 0.01). (C) Heatmap showing the top 50 proteins uniquely and consistently expressed in VC sEVs, ranked based on average expression levels, compared to NDRG1 sEVs ranked by fold change. (D) Heatmap showing the top 20 differentially expressed proteins in sEVs isolated from MIAPaCa‐2 VC or NDRG1‐overexpressing cells, identified using proteomic mass spectrometry with a library‐free search approach. The first 10 proteins (top rows) are more highly expressed in VC sEVs, while proteins ranked 11–20 (bottom rows) are more highly expressed in NDRG1 sEVs. (E) t‐Distributed Stochastic Neighbour Embedding (t‐SNE) plot showing clustering of proteomic profiles from sEVs isolated from MIAPaCa‐2 VC or NDRG1‐overexpressing cells. Each point represents an independent replicate (*n* = 3), with VC sEVs (red) and NDRG1 sEVs (blue). (F) Heatmap showing the expression levels of EV markers (ALIX, CD9, TSG101, CD81, CD63, ANXA5, and ARF6) in 10K EVs and sEVs isolated from MIAPaCa‐2 VC or NDRG1‐overexpressing cells. (G) Heatmap showing the top 20 differentially expressed proteins in 10K EVs isolated from MIAPaCa‐2 VC or NDRG1‐overexpressing cells. The first 10 proteins (top rows) are more highly expressed in VC 10K EVs, while proteins ranked 11–20 (bottom rows) are more highly expressed in NDRG1 10K EVs. (H) Volcano plot representing differentially expressed proteins in 10K EVs isolated from MIAPaCa‐2 cells. Proteins significantly upregulated in VC 10K EVs are shown on the left, while those upregulated in NDRG1 10K EVs are shown on the right. Red dots indicate proteins with significant differential expression (*p* < 0.01, Statistical significance was determined using Student's *t*‐test). (I) Heatmap displaying proteins uniquely and consistently expressed in 10K EVs from MIAPaCa‐2 VC or NDRG1‐overexpressing cells. In all heatmaps, each column represents an independent replicate (#1, #2, #3). All data were normalized and log10‐transformed, with yellow indicating higher expression and blue indicating lower expression and black indicating no expression. Statistical significance was determined using Student's *t*‐test.

Examining sEVs, our analysis revealed a total of 3002 proteins across both conditions, with 2331 proteins detected in sEVs from both cell types. A striking asymmetry emerged in the distribution of unique proteins, with VC sEVs containing 575 unique proteins, whereas NDRG1 sEVs contained only 96 unique proteins. This marked reduction in protein diversity suggests that NDRG1 significantly restricts the range of cargo packaged into sEVs (Figure 3A).

Statistical analysis identified distinct protein signatures that distinguished NDRG1 sEVs from their VC counterparts. Transport‐related proteins such as SLC31A1 (copper transporter) and amino acid transporter SLC7A5, along with metabolic enzymes including phosphoglycerate mutase 1 (PGAM1) (Luo et al. 2023), isocitrate dehydrogenase 1 (IDH1) (Yang et al. 2021), and galactokinase 1 (GALK1) (Mangini et al. 2024), were significantly enriched in VC sEVs (Figure 3B). In contrast, NDRG1 sEVs showed elevated levels of cytoskeletal regulatory proteins, including moesin (MSN), ezrin (EZR), and cadherin‐related protein cullin‐associated and neddylation‐dissociated 1 (CAND1), as well as signalling molecules such as biliverdin reductase A (BLVRA) and hit protein 1 (HINT1) (Figure 3B).

A heatmap of differentially and uniquely expressed proteins in VC sEVs (Figure 3C) highlighted factors involved in TGF‐β signalling (TGFβ1, TGFBR1, BMPR1A) (Heldin and Moustakas 2016), immune modulation (CD276, IGKV3‐20, IL13RA1, LTBR) (Martorelli et al. 2012; Dey et al. 2020; Xu et al. 2021), matrix remodelling (MFAP3, THBS3), and intracellular trafficking/autophagy (ATG9A, SNX27, SPPL2A, VAMP2) (Yang et al. 2011; Steinberg et al. 2013; Mentrup et al. 2020; Mendez et al. 2011). Iron metabolism related heme export and cholesterol/lipid metabolic regulators (FLVCR1, LIPA) (Philip et al. 2015; Beloribi‐Djefaflia et al. 2016) and signal transduction mediators (PTPRJ, PTPRS [Ruckert et al. 2019]) were also enriched in VC sEVs. Further, NOTCH3, which is one of the most studied CAF activating factors (Song and Zhang 2018), was only found in VC sEVs, indicating that VC‐derived sEVs carry distinct functional cargo compared to those from NDRG1‐overexpressing cells. In contrast, only two proteins were found to be consistently and uniquely expressed in all three independent replicates of sEVs derived from NDRG1‐overexpressing cells, including calcium binding protein CALB2 and mitochondrial proteins GATD3A/B (Figure 3C).

Among the most DEPs, the membrane protein SERINC1 showed the highest upregulation in VC sEVs, followed by HSPG2, TGOLN2, H2BU1, and TNFRSF10B. Notably, the glycoprotein GPC1, previously reported as a PaC sEVs marker (Jin et al. 2023; Melo et al. 2015), was significantly enriched in VC sEVs. In contrast, cytoskeletal regulators MSN, EZR, and RDX demonstrated substantially higher abundance in NDRG1 sEVs across all replicates (Buenaventura et al. 2023) (Figure 3D). Dimensionality reduction analysis using t‐SNE demonstrated clear separation between VC and NDRG1 sEV proteomes (Figure 3E), confirming that NDRG1 expression substantially reshapes the overall sEV protein landscape.

To confirm the identity and purity of our EV preparations, we also examined the distribution of canonical EV markers across both 10K EV and sEVs fractions (Figure 3F). As expected, sEV membrane tetraspanins (CD9, CD81, CD63) and ESCRT‐associated proteins (ALIX, TSG101; [Mathieu et al. 2021]) were predominantly detected in the sEV fractions, while the 10K EV fractions showed higher expression of EV markers such as ANXA5 and ARF6 (Forero et al. 2024; Sedgwick and D'Souza‐Schorey 2018).

Interestingly, NDRG1's influence on protein cargo extended beyond sEVs to 10K EV as well. In the 10K EV fraction, we observed distinct protein signatures that differentiated EVs derived from VC versus NDRG1‐overexpressing cells. Among the top 10 up‐regulated genes in VC 10K EVs were the antioxidant enzyme SOD1, structural protein KRT4, lipid carrier APOD, metabolic enzyme IDH1, and ceruloplasmin (CP). Conversely, the top 10 up‐regulated genes in NDRG1 10K EVs included hemopexin (HPX), complement component C4A, pregnancy zone protein (PZP), and GDP dissociation inhibitor 2 (GDI2). This distinct pattern of differential protein expression was consistent across all three independent replicates (Figure 3G).

Additional significant alterations in 10K EV cargo included upregulation of mitochondrial tubulins (TUBB8), filamin A (FLNA), transglutaminase 2 (TGM2), and interferon regulatory factor (IFRH2) in VC 10K EVs, while annexin A6 (ANXA6), ubiquilin‐2 (UBQLN2), basic transcription factor 3 (BTF3), and 2'‐deoxynucleoside 5'‐phosphate N‐hydrolase 1 (DNPH1) were elevated in NDRG1 10K EVs (Figure 3H). Finally, we also observed that NDRG1 10K EVs had some exclusively expressed proteins that were not detected in VC 10K EVs (Figure 3I), including calcium binding proteins, calbindin 2 (CALB2) and S100 calcium binding protein A2 (S100A2), cytoskeleton remodelling proteins and cell adhesion proteins, such as EPS8 Like 2 (EPS8L2) and fermitin family member 1 (FEMT1). In contrast, the chromatin remodelling protein CHD8 was exclusively expressed in the VC 10K EVs (Figure 3I).

Collectively, our comprehensive proteome analysis demonstrates that NDRG1 expression substantially alters the protein cargo profiles of both sEVs and 10K EVs in MIAPaCa‐2 cells. These proteomic changes involve proteins associated with multiple cellular processes, including signalling pathways, metabolism, cytoskeletal organization, and vesicular trafficking, suggesting that NDRG1 broadly influences the composition of EVs secreted by PaC cells.

## 4 Small EVs derived From NDRG1‐Overexpressing PaC Cells Attenuate PSC Activation

**FIGURE 4:**
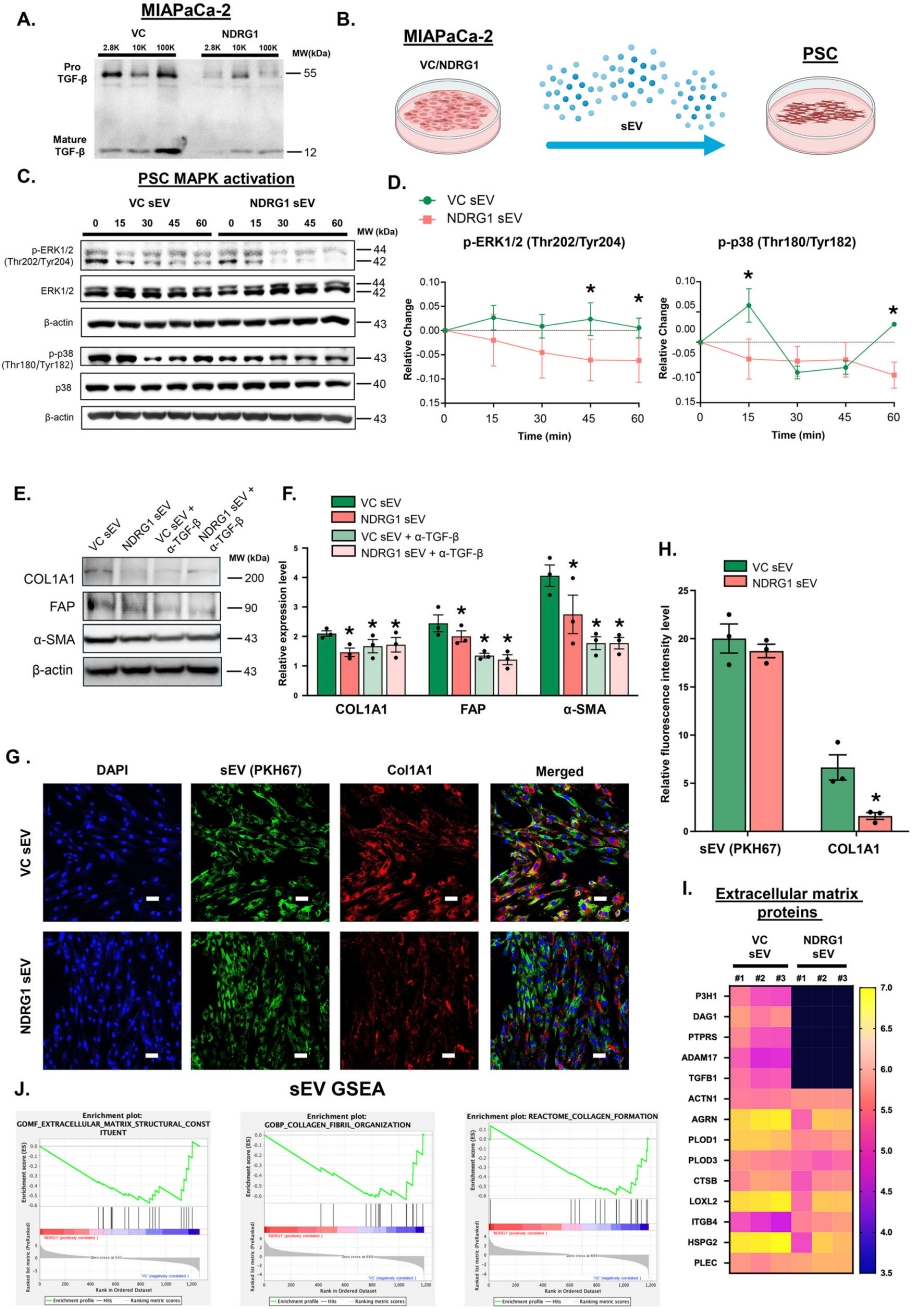
NDRG1‐derived sEVs reduce PSC activation and collagen production compared to VC‐derived sEVs. (A) Representative Western blot images showing the levels of pro‐TGF‐β and mature TGF‐β in EV fractions (2.8K, 10K, and 100K) isolated from VC or NDRG1 MIAPaCa‐2 cells. (B) Schematic illustrating the experimental setup for sEV treatment. (C) Representative Western blot images of MAPK pathway activation in PSCs treated with equal amounts of sEV from VC or NDRG1‐overexpressing MIAPaCa‐2 cells for the indicated time points (0, 15, 30, 45, 60 min). Levels of phosphorylated ERK1/2 (p‐ERK1/2) and p38 (p‐p38) were assessed relative to total ERK1/2 and p38, respectively, with β‐actin serving as a loading control. (D) Quantification of relative changes in p‐ERK1/2 and p‐p38 phosphorylation over time, normalized to total ERK1/2 and p38, respectively. Asterisks (*) indicate significant expression differences between VC and NDRG1 sEV treated PSCs (*n* = 3) (E) Representative Western blot images of CAF activation markers (COL1A1, FAP, and α‐SMA) in PSCs following 24 h treatment with sEVs isolated from VC or NDRG1‐overexpressing MIAPaCa‐2 cells. Treatments were performed with or without pre‐incubation with a TGF‐β neutralizing antibody (α‐TGF‐β). β‐actin was used as a loading control (*n* = 3). (F) Densitometric analysis of protein expression levels normalized to β‐actin. (G) Representative immunofluorescence images of PSCs treated with sEVs from VC or NDRG1‐overexpressing cells for 24 h. Nuclei were stained with DAPI (blue), sEVs are labelled with PKH67 (green), and COL1A1 (collagen type I; red) marks PSC collagen production. Scale bar: 40 µm. (H) The bar graph quantifies fluorescence intensity levels of sEV uptake (PKH67) and COL1A1 expression from G (*n* = 3). (I) Heatmap showing the significantly differentially expressed extracellular matrix (ECM) proteins in sEVs isolated from MIAPaCa‐2 VC or NDRG1‐overexpressing cells. Each column represents an independent replicate (#1, #2, #3). Data were normalized and log10‐transformed, with yellow indicating higher expression and blue indicating lower expression and black indicating no expression. (J) Gene set enrichment analysis (GSEA) plots illustrate the enrichment of ECM‐related pathways in sEV isolated from MIAPaCa‐2 VC or NDRG1‐overexpressing cells, based on proteomic mass spectrometry data. The analysed pathways include GOMF_Extracellular Matrix Structural Constituent, GOBP_Collagen Fibril Organization, and REACTOME_Collagen Formation. In all bar graphs, each dot represents an independent experiment, with bars indicating mean ± SEM. Statistical significance was determined using Student's *t*‐test and is indicated as *p < 0.05.

The proteomic analysis above revealed that TGFβ1 signalling components were reduced in sEVs from NDRG1‐overexpressing cancer cells (Figure 3C). Immunoblot analysis confirmed markedly reduced TGFβ1 expression in NDRG1 sEVs compared to VC sEVs, with this reduction being consistent across all EV fractions (2.8K, 10K, and 100K) derived from NDRG1‐overexpressing cells (Figure 4A). Both precursor (55 kDa) and mature (12 kDa) forms of TGFβ1 showed lower abundance in NDRG1‐derived sEVs. Given the key role TGFβ1 plays in driving PSC activation and ECM deposition in the TME (Wu et al. 2021, Curtis et al. 2019), we investigated the functional consequences of this altered sEVs cargo on PSC behaviour by incubating PSCs with sEVs isolated from either VC or NDRG1‐overexpressing MIAPaCa‐2 cancer cells (Figure 4B). As VC cells secreted approximately 25% more sEVs than NDRG1‐overexpressing cells (Figure 1B), we adjusted the VC‐derived sEV input by 20% prior to downstream assays to approximate equivalent particle dosing between VC and NDRG1‐derived sEVs. Subsequent NTA analysis confirmed that particle concentrations were more closely aligned following this adjustment, with a residual difference of approximately 14% between conditions (Figure S1A).

To examine signalling changes in PSCs after sEV uptake, a human phospho‐kinase array was performed. PSCs treated with sEVs from VC or NDRG1‐overexpressing MIAPaCa‐2 cells showed a notable difference in kinase activation profiles between the two treatment groups. Specifically, the phosphorylation of several critical components of the MAPK signalling pathway, a key regulator of cellular proliferation, differentiation, and stress responses (Kumar et al. 2023; Melchionna et al. 2023) were found to be markedly downregulated in PSCs treated with sEVs from NDRG1‐overexpressing cancer cells (Figure S5A,B). These proteins include ERK1/2 and p38a, which have previously been implicated to induce activation of PSCs into CAFs (Liu et al. 2025; Yan et al. 2019; Kiss et al. 2015). Time‐course analysis of PSCs exposed to cancer cell‐derived sEVs revealed distinct phosphorylation patterns of ERK1/2 (Thr202/Tyr204) and p38α (Thr180/Tyr182) between sEV treatment groups (Figure 4C,D). PSCs treated with sEVs derived from NDRG1‐overexpressing cells exhibited significantly reduced ERK1/2 phosphorylation at 45 and 60 min compared to those treated with VC sEVs (Figure 4C,D). Similarly, p38α phosphorylation was significantly lower in PSCs treated with sEVs derived from NDRG1‐overexpressing cells at 15 and 60 min timepoints (Figure 4C,D). Total ERK1/2 and p38α levels remained unchanged throughout the incubation period confirming that the observed differences reflected altered activation rather than protein abundance.

To determine whether the attenuated MAPK signalling was linked to reduced TGF‐β content in NDRG1‐overexpressing sEVs and how this affected PSC differentiation into CAFs, we performed a TGF‐β neutralization study. PSCs were incubated with either VC‐derived sEVs or NDRG1‐derived sEVs in both the absence and presence of TGF‐β neutralizing antibody (α‐TGF‐β), and expression of CAF activation markers examined. In the absence of the neutralizing antibody, PSCs treated with sEVs derived from NDRG1‐overexpressing cancer cells exhibited significantly lower levels of classical CAF markers, including COL1A1, FAP, and α‐SMA, compared to PSCs treated with VC‐derived sEVs (Figure 4E,F). Further, a similar reduction in these CAF‐activation markers was also observed in PSCs following incubation with VC‐derived sEVs that had the α‐TGF‐β added. Addition of α‐TGF‐β to NDRG1‐derived sEVs did not further reduce the CAF activation markers when compared to NDRG1‐derived sEVs that did not contain the α‐TGF‐β (Figure 4E,F). Overall, these results support the hypothesis that NDRG1 expression in PaC cells interrupts PSC activation, possibly by disrupting the sEV cargo loading machinery and reducing its TGF‐β cargo (Geleta et al. 2021).

To validate these findings at the cellular level, we performed immunofluorescence microscopy on PSCs incubated with fluorescently labelled (PKH67) sEVs from either VC or NDRG1‐overexpressing MIAPaCa‐2 cells for 24 h (Figure 4G). SEVs from both VC and NDRG1‐overexpressing cancer cells were taken up by PSCs at similar rates, as demonstrated by the green fluorescence from the PKH67 dye accumulating in PSCs (Figure 4G). However, the expression of COL1A1 (Collagen type I alpha 1; red signal) was dramatically reduced in PSCs exposed to NDRG1 sEVs. Quantification of fluorescence intensity confirmed significant collagen reduction, with approximately 80% lower COL1A1 expression in NDRG1 sEV‐treated PSCs compared to the VC group (Figure 4H). As sEV uptake itself was not significantly different between conditions, this suggests that the observed effects stemmed from altered sEV cargo rather than differential uptake efficiency.

Given the decrease in collagen production, we next examined whether this reflected broader changes in the extracellular‐matrix cargo of NDRG1‐derived sEVs. Heatmap analysis revealed differential expression of multiple ECM‐associated proteins between NDRG1 and VC sEVs across all three independent replicates (Figure 4I). Several key ECM components, including prolyl 3‐hydroxylase (P3H1) (Li et al. 2024), dystroglycan 1 (DAG1 [Quereda et al. 2022]), protein tyrosine phosphatase receptor type S (PTPRS), and ADAM metallopeptidase 17 (ADAM17), were not detected in NDRG1 sEVs. Moreover, peptides such as Agrin (AGRN) (Mazzon et al. 2011), procollagen‐lysine,2‐oxoglutarate 5‐dioxygenase 1 (PLOD1) (Zhai et al. 2024), procollagen‐lysine,2‐oxoglutarate 5‐dioxygenase 3 (PLOD3), cathepsin B (CTSB), lysyl oxidase‐like 2 (LOXL2) (Torres et al. 2015; Tanaka et al. 2018), and heparan sulfate proteoglycan 2 (HSPG2) exhibited significantly higher expression levels in VC derived sEVs compared to sEVs derived from NDRG1‐overexpressing cancer cells. Gene Set Enrichment Analysis (GSEA) of the proteomic data provided further evidence of functional divergence between sEV populations. Pathways related to ECM organization and collagen synthesis, specifically “ECM structural constituent,” “collagen fibril organization,” and “collagen formation”, were significantly enriched in VC sEVs (Figure 4J), with pronounced enrichment scores and distinct clustering of ECM‐related genes.

Together, these results demonstrate that sEVs from NDRG1‐overexpressing cancer cells disrupt TGFβ1‐mediated MAPK activation and substantially reduce CAF marker expression and collagen production in PSCs, highlighting NDRG1's potential role in modulating tumour‐stroma interactions through altered sEVs communication.

## 5 NDRG1 Reduces sEVs Uptake and Promotes sEVs–Lysosome Interactions

To extend our understanding of how NDRG1 modulates cross‐talk between PaC cells and the TME, we also investigated whether NDRG1 influences cancer cell uptake of sEVs. SEVs can serve as nutrient sources under metabolic stress, and previous evidence suggests PaC cells may “rewire” nearby CAFs to supply metabolite‐rich sEVs (Yang et al. 2020). Our earlier observations (Figure 1G) showed that Rab5a, a key protein regulating early endosomal trafficking (Bucci et al. 1994), was downregulated in NDRG1‐overexpressing cancer cells. This prompted us to examine whether NDRG1 can also influence sEV uptake by PaC cells.

**FIGURE 5:**
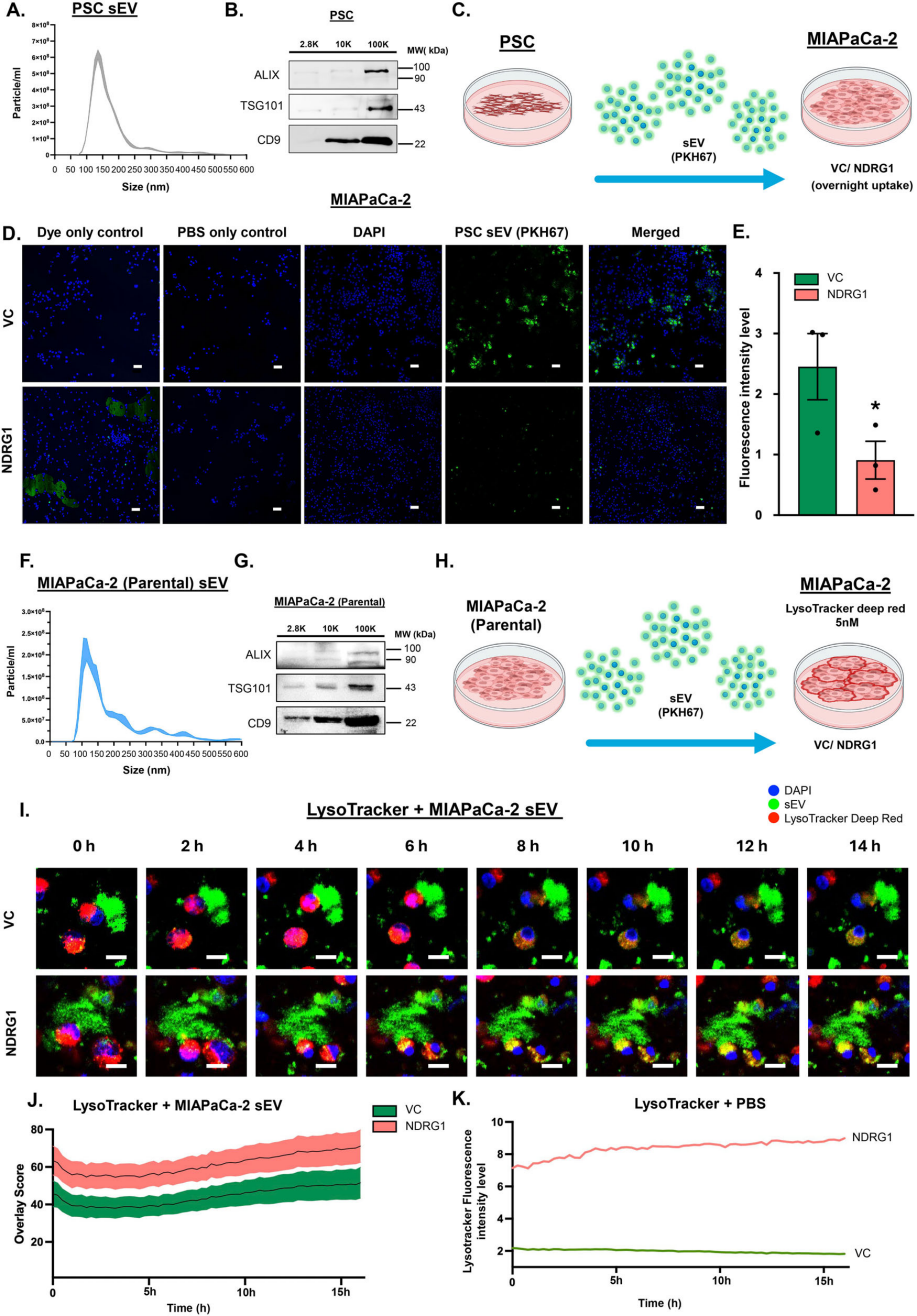
NDRG1 decreases sEVs uptake from the TME and directs internalized sEVs to the lysosome. (A) NTA showing the size distribution of PSC‐derived 100K sEVs. (B) Representative Western blot images of sEV markers (ALIX, TSG101, and CD9) in PSC‐derived EVs confirming the presence of sEV markers in the 100K fraction. (C) Schematic illustrating the experimental setup where pancreatic stellate cells (PSCs) secrete sEV, which are labelled with PKH67 (green) and subsequently incubated overnight with MIAPaCa‐2 VC or NDRG1‐overexpressing cells. (D) Representative immunofluorescence images of VC or NDRG1‐overexpressing MIAPaCa‐2 cells incubated with dye only control, PBS only control or PSC‐derived sEVs overnight. Nuclei are stained with DAPI (blue), and PKH67‐labeled PSC sEVs are green. Merged images indicate sEV uptake. Scale bar: 60 µm. (E) Quantification of sEV uptake fluorescence intensity in NDRG1‐expressing MIAPaCa‐2 cells compared to VC cells. Each dot represents an independent experiment (*n* = 3), with bars indicating mean ± SEM. Statistical significance was determined using Student's *t*‐test, *p < 0.05. (F) NTA showing the size distribution of parental MIAPaCa‐2‐derived 100K sEVs. (G) Representative Western blot images of sEV markers (ALIX, TSG101, and CD9) in parental MIAPaCa‐2‐derived EVs confirming the presence of sEV markers in the 100K fraction. (H) Schematic illustrating the experimental setup where sEVs from MIAPaCa‐2 parental cells (PKH67‐labeled, green) were added to VC and NDRG1‐overexpressing MIAPaCa‐2 cells pre‐treated with LysoTracker Deep Red (5 nM) and Hoechst live nuclei stain (2 µg/mL) for 1 h, which is followed by a 2.5 h incubation with sEVs to ensure uptake prior to imaging. (I) Representative time‐lapse confocal microscopy images showing sEV uptake and trafficking in VC and NDRG1‐expressing MIAPaCa‐2 cells over time. DAPI (blue) stains nuclei, PKH67 (green) marks sEVs, and LysoTracker Deep Red (red) labels lysosomes. Confocal live‐cell imaging was performed with Leica Stellaris 8 for 16 h at 37°C and 5% CO_2_, capturing 16 ROIs/sample every 15 min (total 65 time points). Yellow colour indicate colocalization of sEVs with lysosomes. Scale bar: 20 µm. (J) Quantification of colocalization (overlap score) between sEVs and lysosomes in VC and NDRG1‐expressing cells, calculated using the Fiji Multiply function. The overlap score represents the degree of sEVs‐lysosome colocalization over time. The shaded area represents the standard SEM at every timepoint. (K) Quantification of LysoTracker fluorescence intensity over time in control conditions (PBS‐treated cells). Fluorescence intensity and overlay score were normalized to the intensity of the nucleus.

SEVs were enriched from PSC conditioned media, with NTA and immunoblotting confirming particle size and marker expression was consistent with sEVs (Figure 5A,B). Small EVs were stained with PKH67 dye and loaded onto both MIAPaCa‐2 VC and NDRG1‐overexpressing cells, followed by an overnight incubation (Figure 5C). Confocal immunofluorescence revealed significantly fewer PKH67‐labelled sEVs were detected in NDRG1‐overexpressing cells compared with VC cells (Figure 5D,E). This was further validated using PANC‐1 cells, where the NDRG1‐overexpressing PANC‐1 cells internalized significantly fewer PSC‐derived sEVs (Figure S5C,D).

To further validate this result, an orthogonal protein‐based labelling strategy using an amine‐reactive fluorescent NHS ester (Ester 488) was also used to label PSC‐derived sEVs prior to their incubation with MIAPaCa‐2 VC and NDRG1‐overexpressing cells. Similar to what was observed with PKF67‐labelled sEVs (Figure 5D,E), the uptake of Ester 488‐labelled sEVs were also significantly reduced in NDRG1‐overexpressing cells when compared to the VC cells (Figure S5E,F). Together, these results demonstrate that increased NDRG1 expression in PaC cells reduces their uptake of PSC‐derived sEVs.

We next explored whether NDRG1 also modulates the intracellular fate of internalized sEVs. Our earlier findings (Figure 1G) and previous research (Menezes et al. 2019; Ortega et al. 2019), indicate that NDRG1 can regulate lysosomal function, raising the possibility that TME‐derived sEVs might be redirected for lysosomal degradation in NDRG1‐overexpressing cells. To investigate this, we isolated and validated sEVs from parental MIAPaCa‐2 cells (Figure 5F,G), followed by labelling with PKH67 dye. MIAPaCa‐2 VC or NDRG1 cells were pre‐incubated with lysosomal stain LysoTracker Deep Red, followed by incubation with the PKH67‐labelled sEVs (Figure 5H). Live‐cell confocal imaging was then performed over 16 h under standard cell culture conditions to track whether internalized sEVs were co‐localizing with lysosomes (Figure 5I).

Colocalization measurements confirmed a significantly greater overlap between PKH‐labelled sEVs (green) and lysosomes (red) in the NDRG1‐overexpressing cells, as demonstrated by the increased abundance of yellow puncta (Figure 5I) and reflected by the higher overlay score of the two markers (Figure 5J). Analysis of the confocal images also indicated a significant increase in basal lysosomal activity over the course of 16 h in NDRG1‐overexpressing MIAPaCa‐2 cells relative to VC cells (Figure 5K), consistent with our earlier observations of elevated LAMP2 in NDRG1‐overexpressing PaC cells (Figure 1H). These data suggest that NDRG1 not only reduces the number of sEVs taken up by cancer cells, but appears to direct them towards the lysosomes, potentially limiting the metabolite or signalling advantages these sEVs confer within the TME.

## 6 Discussion

It has been well established that the TME is a key contributor to PaC progression and development of resistance to current therapies. However, targeting the TME in PaC has proven challenging due to its dynamic nature that is constantly being reshaped by both cancer and stromal cells. This is mediated by the bi‐directional cross talk between cancer and stromal cells, which exchange EVs, growth factors, metabolites and cytokines to reprogram the function of neighbouring cells and manifest a favourable niche for PaC cells to proliferate and metastasise (Hosein et al. 2020; Derle et al. 2018). In the current study, we demonstrate for the first time that the metastasis suppressor NDRG1 can inhibit the cellular communication between PaC cells and the TME, with this effect being mediated by disruption of sEV release, biogenesis, cargo packaging and uptake by cancer cells. NDRG1 significantly attenuated sEV release from PaC cells, an effect that was likely due to the ability of NDRG1 to inhibit sEV biogenesis machinery by potentially interacting with and regulating the expression of various ESCRT proteins, including HRS, TSG101, ALIX, and CD9. NDRG1 reduced the expression of the ESCRT‐0 protein TSG101, which is involved in ESCRT initiation, maturation of early and late endosomes and MVB cargo sorting (Schmidt and Teis 2012). This suggests that NDRG1 expression can inhibit the initiation of sEV biogenesis potentially by regulating the ESCRT‐0 complex.

ALIX is another important ESCRT pathway regulator and a key component of ESCRT‐III that is predominantly involved in late endosomal and MVB cargo selection (Martin‐Serrano et al. 2003; Baietti et al. 2012; Roucourt et al. 2015). We demonstrate that NDRG1 down‐regulates ALIX expression, with this effect potentially being arbitrated through ubiquitin‐mediated proteasomal degradation. We also demonstrate that a knock‐down of ALIX in the MIAPaCa‐2 cells reduced sEV secretion, suggesting that the NDRG1‐mediated effects on sEV secretion are mediated by ALIX. In fact, previous studies have shown that ALIX facilitates sEV cargo sorting, secretion, and drives malignant phenotypes in transformed cancer cells (Baietti et al. 2012; Hikita et al. 2019). Furthermore, ALIX‐dependent sEV secretion has been linked to tumour immune evasion and metastasis, as it is involved in packaging immune‐regulatory proteins such as PD‐L1 into sEVs, thereby modulating anti‐tumour immunity (Monypenny et al. 2018). A number of earlier studies have also shown that NDRG1 can regulate the expression of other proteins by facilitating their ubiquitination and subsequent proteasomal degradation in pancreatic and other cancers (Chekmarev et al. 2021; Hoshino et al. 2024; Yang et al. 2017). This suggests that NDRG1 might have a broader functional effect on the proteasomal degradation machinery, a hypothesis that is further supported by the increased turnover of ubiquitin observed in the NDRG1‐overexpressing PaC cells in this study.

Notably, besides promoting ALIX ubiquitination and eventual degradation, the direct binding of NDRG1 to ALIX may also inhibit its function in the first instance. ALIX and its interaction with ESCRT‐III, is crucial for ILV formation and protein sorting during MVB maturation (Bissig and Gruenberg 2014; Larios et al. 2020). The predicted spatial interaction between NDRG1 and ALIX, particularly at the Bro1 domain which mediates ESCRT‐III recruitment, suggests that NDRG1 may interfere with critical protein‐protein interactions required for MVB formation. This mechanism differs from previously described regulators of sEV biogenesis that typically act through transcriptional control of ESCRT components or modulation of endosomal trafficking without direct protein‐protein interactions (Hoffman et al. 2019).

In addition to ESCRT pathway proteins, we also found that CD9, a surface marker for sEVs, was significantly upregulated in PaC cell lines by NDRG1. Interestingly, CD9 upregulation was previously related to increased lysosome number, while deletion of CD9 promoted MVB formation and sEV secretion in melanoma cancer cells (Suárez et al. 2021). These findings demonstrate that the ESCRT complex is intimately associated with endo‐lysosomal vesicular structure formation and maturation, and that NDRG1 might influence both intracellular processes.

This was further confirmed by our observations that NDRG1 altered the expression of major regulators of endo‐lysosomal activities, namely the Rab GTPase family members Rab5a, Rab27a and Rab9a. Work by Ostrowski et al. identified that reduced levels of either Rab27a/b, or Rab5a can lead to decreased sEV secretion (Gorji‐bahri et al. 2021; Ostrowski et al. 2010). However, these different Rab GTPases oversee different parts of intracellular vesicular transportation. For instance, Rab27a is the “docking‐and‐fusion” specialist positioned at the cell periphery, while Rab27b shepherds membranes from the trans‐Golgi network (TGN) toward MVEs, priming them for subsequent Rab27a‐dependent fusion (Ostrowski et al. 2010). Rab5a is known as a key endosomal trafficking regulator that mainly controls endocytosis and early endosome formation (Nagano et al. 2019; Saitoh et al. 2017; Gulappa et al. 2011). Hence, our observation that NDRG1 overexpression reduced cancer cell sEV secretion and uptake, can also be explained by impaired MVB maturation and failed initiation of endocytosis due to the alterations in these Rab GTPases.

In agreement with our observations, others have also shown that NDRG1 may regulate EV biogenesis through its interactions with vesicular trafficking machinery. NDRG1 can directly interact with Prenylated Rab Acceptor 1 protein (PRA1), which then regulates small GTPase Rab proteins that facilitate transport between cell organelles (Hunter et al. 2005). Moreover, evidence suggests that NDRG1 directly binds to Rab4a to regulate E‐cadherin transport and recycling through an endosome‐related mechanism (Kachhap et al. 2007). In addition, NDRG1‐silenced cells were found to have impaired lysosomal function and increased secretion of neutral sphingolipids and ceramides (Pietiäinen et al. 2013; Ortega et al. 2019; Sevinsky et al. 2018), which are known to affect sEV biogenesis (Trajkovic et al. 2008). This is consistent with our findings in MIAPaCa‐2 cells where NDRG1 overexpression resulted in increased levels of lysosomal marker LAMP‐2 and Rab9a, which is involved in lysosome formation (Mahanty et al. 2016; Eskelinen 2006). Interestingly, there is evidence suggesting that NDRG1 also regulates lysosome and proteasome activities (Menezes et al. 2019; Ortega et al. 2019; Yang et al. 2017). Ortega et al. discovered that NDRG1 silencing increased sEVs release in breast, colon and ovarian cancer cell lines, with this effect being accompanied by inhibition of lysosomal acidification (Ortega et al. 2019). The abovementioned effects of NDRG1 on lysosomal degradation of numerous proteins may potentially be mediated by its effects on or association with Rab9a.

Another important finding of this study is the alteration of sEV protein cargo in NDRG1‐overexpressing cells. Our study reveals that NDRG1 expression reshapes the EV proteome in PaC cells, selectively restricting the packaging of pro‐tumourigenic, metabolic, and stromal‐activating proteins. Through comprehensive proteomic analysis of both sEVs and 10K EVs, we show that NDRG1 narrows the range of vesicular cargo and actively modulates intercellular communication in a manner consistent with its well‐established role as a metastasis suppressor (Kovacevic and Richardson 2006; Mi et al. 2017; Sun et al. 2013). One of the most striking findings was the reduction in the diversity of sEV proteins secreted by NDRG1‐overexpressing cells compared to VC. A global proteomic analysis revealed a 16.5% reduction in total protein content in NDRG1‐derived sEVs (2472 proteins) compared to VC controls (2906 proteins). This demonstrates the capacity of NDRG1 to reduce the dissemination of signalling molecules via the EV pathway. This reduction likely limits the tumour cells’ ability to interact with and modify the TME, representing a novel mechanism by which NDRG1 enforces a less aggressive cellular phenotype.

Importantly, TGFβ1 levels were reduced in NDRG1‐derived sEVs, as determined by Western blotting, and multiple TGFβ pathway components (including TGFβ1, TGFBR1, TGFBR3, and BMPR1A) were below the detection threshold of the proteomic analysis. In contrast, these proteins were abundant in VC‐derived sEVs, suggesting a selective suppression of TGFβ‐mediated tumour–stroma crosstalk. TGF‐β family members are well established drivers of PSC activation, promoting the dense desmoplastic stroma characteristic of pancreatic tumours and contributing to metastasis and therapy resistance (Costa‐Silva et al. 2015; Binang et al. 2023). Consistent with this, the proteomic analysis indicated that VC‐derived sEVs were enriched in NOTCH3, a key regulator of cancer‐associated fibroblast activation that promotes fibrosis, immune suppression, and tumour progression (Ghosh and Mitra 2023). In contrast, NOTCH3 was below the detection threshold in NDRG1‐derived sEVs. Together, these findings support a model in which NDRG1 limits stromal activation and remodelling through selective depletion of pro‐fibrotic signalling cargo from sEVs.

Consistent with a role in suppressing migration and invasion, our proteomic analysis revealed that NDRG1‐overexpressing cells secreted sEVs enriched in cytoskeletal regulators such as EZR, RDX and MSN (ERM proteins). The export of these actin‐binding proteins, critical mediators of membrane‐cytoskeleton dynamics, may serve to stabilize epithelial architecture internally while shedding pro‐migratory potential externally (Tsukita 1997; Gautreau et al. 2002). This model aligns with previous findings that NDRG1 promotes epithelial integrity and inhibits EMT (Kovacevic and Richardson 2006; Mi et al. 2017; Sun et al. 2013), although a direct link to the ERM proteins requires further validation.

Our proteomic analysis further demonstrated that NDRG1 expression altered vesicle‐associated calcium signalling components. Proteins such as CALB2 and S100A2, both involved in calcium buffering and modulation of calcium‐dependent signalling pathways, were selectively enriched in NDRG1 10k large EVs. Given that calcium signalling governs numerous aspects of cancer biology, including proliferation, apoptosis, and migration (Prevarskaya et al. 2011), the controlled export of calcium regulatory proteins may help maintain calcium homeostasis within NDRG1‐expressing cells, while modulating calcium‐sensitive pathways in recipient cells. Interestingly, prior studies have also suggested that NDRG1 can directly regulate intracellular calcium signalling, influencing processes such as stress response and cytoskeletal organization (Merlot et al. 2019; Stein et al. 2004). Moreover, new evidence indicates that CALB2 is not a passive calcium buffer, rather it actively drives inflammatory reprogramming and immunosuppression in PaC by promoting a Ca^2^^+^‐CXCL14 inflammatory axis that enhances metastatic outgrowth (Tao et al. 2024). High CALB2 expression in CAFs and cancer cells correlates with poor prognosis and immunosuppressive TME. These findings raise intriguing questions about the biological meaning of EV‐mediated secretion of CALB2 and its impact on the surrounding microenvironments, with further investigation warranted.

An additional and critical layer of EV cargo reprogramming by NDRG1 involves metabolic crosstalk. Small EVs derived from VC cells were enriched in key metabolic enzymes such as IDH1, PGAM1, GALK1, as well as nutrient transporters including SLC31A1 and SLC7A5. IDH1 is a critical enzyme in the tricarboxylic acid (TCA) cycle and has been shown to contribute to redox balance and promote cancer cell adaptation under metabolic stress conditions (Ward et al. 2010). PGAM1, a key glycolytic enzyme, supports rapid tumour growth by enhancing glycolytic flux and promoting anabolic biosynthesis (Hitosugi et al. 2012). GALK1 regulates galactose metabolism, which can be diverted toward glycoprotein and glycolipid biosynthesis pathways essential for tumour cell surface remodelling (Brockhausen 2006). Moreover, SLC31A1 facilitates copper uptake, a trace element required for angiogenesis and mitochondrial function in cancer (Blockhuys and Wittung‐Stafshede 2017), while SLC7A5 functions as a major amino acid transporter necessary for mTORC1 activation and metabolic reprogramming in tumours (Nicklin et al. 2009). Together, these proteins may enable metabolic reprogramming of the TME by supplying biosynthetic intermediates, enhancing nutrient uptake, and reshaping redox balance in recipient stromal and immune cells (Kalluri 2016). The exclusion of these metabolic regulators from NDRG1 EVs suggests inhibition of tumour‐promoting metabolic symbiosis, further supporting the metastasis‐suppressor role of NDRG1.

Interestingly, NDRG1 sEVs selectively incorporated BLVRA, an enzyme intimately involved in heme catabolism, antioxidant defence, and iron metabolism (Foresti and Motterlini 1999; Lei et al. 2024). BLVRA activity reduces biliverdin to bilirubin, a potent antioxidant that mitigates reactive oxygen species (ROS) accumulation, and its activity is tied to maintaining iron homeostasis (Foresti and Motterlini 1999; Lei et al. 2024; Gibbs et al. 2015; Kubícková et al. 2016). This observation is especially significant given that NDRG1 is known to be transcriptionally upregulated in response to iron chelation and modulates intracellular iron levels (Geleta et al. 2021; Morales and Xue 2021; Jadhav et al. 2024). The selective inclusion of BLVRA in sEVs suggests that NDRG1 may regulate redox and iron stress not only within tumour cells but also in neighbouring stromal and immune cells through vesicle‐mediated signalling.

Building upon the proteomic analysis, which revealed significant changes in protein cargo from NDRG1‐overexpressing PaC cells, we further demonstrate that these molecular alterations have functional consequences on PSCs. Notably, phosphorylation of p38 and pERK1/2, both downstream effectors of the MAPK pathway, was reduced in PSCs following exposure to sEVs from NDRG1‐overexpressing PaC cells. This was accompanied by reduced expression of CAF markers and reduced collagen production, suggesting that NDRG1 expression in PaC cells reduces activation of PSCs into CAFs. This is consistent with our previous reports that NDRG1 overexpression in PaC cells maintains PSCs in a quiescent, non‐fibrogenic state (Geleta et al. 2021), and demonstrates that sEVs are an important messenger for this stromal reprogramming.

The functional impact on ECM remodelling was further reinforced by our proteomic profiling of EV cargo. Several ECM‐related proteins critical for collagen crosslinking and matrix remodelling, such as P3H1, DAG1, PTPRS, and ADAM17, were absent in NDRG1‐derived sEVs. Moreover, multiple ECM remodelling enzymes, including LOXL2, PLOD1, PLOD3, CTSB, and HSPG2, were significantly more abundant in VC‐derived sEVs. GSEA supported these observations, showing enrichment of pathways related to collagen fibril organization and ECM structural constituents exclusively in VC sEVs. These data suggest that NDRG1 expression not only reduces the delivery of soluble profibrotic signals but also limits the transmission of structural ECM components that would otherwise support desmoplasia. These findings further underscore NDRG1's role as a key regulator of tumour–stroma interactions, acting through the strategic modulation of EV communication to potentially reshape the TME toward a less fibrotic and less supportive niche for tumour progression.

In addition, our finding that NDRG1 not only affects cancer cell‐derived sEVs but also modulates the uptake and processing of PSC‐derived sEVs is particularly significant. Recent studies have suggested that cancer cells may “educate” stromal cells to provide metabolite‐rich sEVs that support tumour growth under metabolic stress (Chang et al. 2023; Yang et al. 2020). In the current study, we demonstrate that NDRG1 not only limits the uptake of PSC‐derived sEVs by cancer cells but also directs internalized sEV toward lysosomal degradation, effectively constraining the pro‐tumourigenic advantages conferred by intercellular vesicle transfer. NDRG1's ability to reduce sEV uptake and promote their lysosomal degradation suggests it may block this metabolic support system, potentially contributing to its tumour‐suppressive function.

While our study provides compelling evidence for NDRG1's role in regulating sEV biology in PaC, several questions remain. The precise mechanism by which NDRG1 selectively alters sEV cargo warrants further investigation, particularly regarding the enrichment of certain proteins amid general cargo restriction. Additionally, the effect of NDRG1‐mediated sEV regulation on immune cell interactions within the TME remains to be explored. The demonstrated effect of NDRG1 expression in cancer cells on PSCs in this study is limited to PSCs derived from a single healthy donor, and thus, we cannot rule out potential inter‐donor variability, which would require samples from additional donors to be examined. Finally, whether the NDRG1 effects on PSCs are more potently mediated via EVs or also include secreted factors such as free cytokines/chemokines is yet to be determined. In fact, we have previously demonstrated that NDRG1 can modulate the secretion of several cytokines and signalling molecules such as TGF‐β, Wnt3a and Tenascin‐c by cancer cells (Geleta et al. 2022).

In conclusion, our study establishes NDRG1 as a regulator of sEV biogenesis and intercellular communication in PaC. By interfering with the ESCRT pathway through direct interaction with ALIX, NDRG1 restricts sEV production and reshapes sEV cargo to disrupt oncogenic signalling networks. These results highlight a novel extracellular dimension to NDRG1's metastasis‐suppressor role and suggest that modulating EV cargo selection could represent a new therapeutic strategy in PaC.

## Author Contributions

**Jiawei Chang**: conceptualization, investigation, writing – original draft, formal analysis. **Shafi Alenizi**: resources, formal analysis, investigation. **Heloisa Zaccaron Milioli**: data curation, resources. **Winston Lay**: investigation, resources, formal analysis. **Saranya Pounraj**: data curation, resources. **Yujie Li**: methodology, writing – review and editing. **Mekonnen Sisay Shiferaw**: writing – review and editing, methodology. **Elham Hosseini‐beheshti**: supervision, resources, funding acquisition, writing – review and editing, methodology. **Zaklina Kovacevic**: project administration, supervision, writing – review and editing, funding acquisition, conceptualization.

## Conflicts of Interest

The authors declare no conflicts of interest.

## Supporting information




**Supporting Information**: jev270334‐sup‐0001‐FigureS1–S5.docx

**Supporting Information**: jev270334‐sup‐0001‐FigureS1–S5.docx

## Data Availability

The data that support the findings of this study are available from the corresponding author upon reasonable request.
