# Supplementary material for: Silencing the Signal: The Metastasis Suppressor NDRG1 Disrupts Small Extracellular Vesicle‐Mediated Crosstalk in Pancreatic Cancer
Source: J Extracell Vesicles. 2026 Jun 30;15(7):e70334. doi: 10.1002/jev2.70334 (PMC13317765; doi:10.1002/jev2.70334)
Supplement: Supplementary file 1 — Supporting Information: jev270334‐sup‐0001‐FigureS1–S5.docx [file JEV2-15-e70334-s002.docx]

**Supplemental Figure 1: (A)** NanoSight report comparing MIAPaCa-2 vector control (VC) and NDRG1-overexpressing (NDRG1) cell derived sEV concentration when VC sEVs were reduced by 20%. VC and NDRG1 sEV particle concentrations after adjustment are shown in bold. (**B**) Western blot analysis showing NDRG1 expression in PANC-1 vector control (VC) cells and PANC-1 cells stably transfected to over-express NDRG1 (NDRG1), with β-actin as a loading control. The accompanying densitometry analysis quantifies relative NDRG1 expression, with each dot representing an independent experiment (n=3). **(C)** Nanoparticle tracking analysis (NTA) showing the concentration of small EVs isolated from VC or NDRG1 PANC-1 cells. Each dot represents an independent experiment, and bars indicate the mean ± SEM. The size distribution graph depicts the particle size profile of sEVs measured by NTA, with shaded regions representing the standard error of the mean (SEM), calculated from three independent experiments. (**D**) Representative Western blot images of exosomal markers (ALIX, TSG101, and CD9) and mitochondrial marker (Mitofilin) in 2.8K, 10K, and 100K EV fractions isolated from PANC-1 VC and NDRG1 cells. (**E**) Representative transmission electron microscopy (TEM) images of small EVs isolated from PANC-1 VC and NDRG1 cells. Scale bar: 200 nm. (**F**) Representative Western blot images of small EV markers (ALIX, TSG101, and CD9) in MIAPaCa-2 silence control NDRG1 cells (shCtrl) and NDRG1 silenced cells (shNDRG1). Statistical significance was determined by Student’s *t*-test and is indicated as ***p* < 0.01; ****p* < 0.001.

**Supplemental Figure 2:** (**A**) Representative Western blot images of ESCRT pathway proteins (HRS, ALIX, TSG101, and CD9) and their densitometric analysis in VC or NDRG1 PANC-1 cells. (**B**) Representative Western blot images of endosomal trafficking regulators (Rab27a, Rab27b, Rab5a, Rab9a, and LAMP-2) and their densitometric analysis in VC or NDRG1 PANC-1 cells. (**C**) Representative Western blot images of ESCRT pathway proteins (ALIX, TSG101, and CD9) and their densitometric analysis in MIAPaCa-2 control (shCtrl) cells and MIAPaCa-2 cells stably transfected to knock-down NDRG1 (shNDRG1). (**D**) Representative Western blot images of endosomal trafficking regulators (Rab27a, Rab27b, Rab5a, Rab9a, and LAMP-2) and their densitometric analysis in shCtrl or shNDRG1 MIAPaCa-2 cells. For all densitometric analysis, band intensity was normalized to β-actin. Each dot represents an independent experiment, and bars indicate the mean ± SEM. Statistical significance was determined by Student’s *t*-test and is indicated as **p* < 0.05; ****p* < 0.001.

**Supplemental Figure 3:** **(A)** Representative Western blot images showing ALIX expression in MIAPaCa-2 cells treated with either lipofectamine only (Lipo only), Silencer Select negative control (siCtrl), or 2 different siRNAs targeting ALIX (siALIX #1 and siALIX #2)), with β-actin used as a loading control. The accompanying densitometry analysis quantifies relative ALIX expression, with each dot representing an independent experiment (n=3). **(B)** NTA showing the size distribution graph of sEVs derived from Lipo only, siCtrl, siALIX #1 and siALIX #2. The graph depicts the particle size profile of sEVs measured by NTA, with shaded regions representing the standard error of the mean (SEM) (n=1). (**C**) Representative immunofluorescence images of MIAPaCa-2 cells expressing vector control (VC) or NDRG1, showing staining for NDRG1 (green), Rab9A (red), and nuclei (DAPI, blue), with merged images indicating colocalization of NDRG1 and Rab9A. Scale bar: 20 µm. Quantification of fluorescence intensity levels for NDRG1 and Rab9A in VC and NDRG1 expressing cells is shown in the adjacent bar graph. (**D**) Pearson’s correlation coefficient analysis quantifies the degree of colocalization between NDRG1 and Rab9A. (**E**) Representative immunofluorescence images of MIAPaCa-2 cells expressing VC or NDRG1 showing staining for NDRG1 (red), Rab5A (green), and nuclei (DAPI, blue), with merged images indicating colocalization of NDRG1 and Rab5A. Scale bar: 20 µm. Quantification of fluorescence intensity levels for NDRG1 and Rab5A in VC and NDRG1 expressing cells is shown in the adjacent bar graph. (**F**) Pearson’s correlation coefficient analysis quantifies the degree of colocalization between NDRG1 and Rab5A. (**G**) Representative immunofluorescence images of MIAPaCa-2 cells expressing VC or NDRG1 showing staining for ALIX (red), LAMP-2 (green), and nuclei (DAPI, blue), with merged images. Scale bar: 20 µm. (**H**) Pearson’s correlation coefficient analysis quantifies the degree of colocalization between ALIX and LAMP-2. In all bar graphs, each dot represents an independent experiment, with bars indicating mean ± SEM. Statistical significance was determined using Student’s *t*-test, * *p* < 0.05.

**Supplemental Figure 4: AlphaFold3 Prediction of ALIX and NDRG1 interaction.** (**A**) ALIX shows conformational changes before (blue) and after NDRG1 binding (pink). (**B**) Side view showing NDRG1 binding to the Bro-1 domain of ALIX. (**C**) Binding site between NDRG1 and ALIX shown as surface, while the entire proteins are shown as cartoon.

**Supplemental Figure 5:** (**A**) A Proteome Profiler Human Phospho-Kinase Array (R&D Systems, Cat# ARY003C) was used to assess the phosphorylation status of key kinases in PSCs treated with sEVs from VC or NDRG1-expressing MIAPaCa-2 cells. (**B**) Enlarged representative phospho-kinase array blots from (A) show differential phosphorylation patterns of ERK1/2 (T202/Y204, T185/Y187) and p38α (T180/Y182) in PSCs treated with VC or NDRG1 sEVs. **(C)** Representative immunofluorescence images of VC or NDRG1-expressing PANC-1 cells incubated with PSC-derived sEVs overnight. Nuclei are stained with DAPI (blue), and PKH67-labeled PSC sEVs are green. Merged images indicate sEV uptake. Scale bar: 60 µm. (**D**) Quantification of sEV uptake fluorescence intensity in NDRG1-expressing PANC-1 cells compared to VC cells is shown in the adjacent bar graph. Green fluorescence intensity is normalized to DAPI intensity. Each dot represents an independent experiment, with bars indicating mean ± SEM. Statistical significance was determined using Student’s t-test (*p < 0.05). (**E**) Representative immunofluorescence images of VC or NDRG1-expressing MIAPaCa-2 cells incubated with a dye-only control, PBS-only control, or PSC-derived small EVs overnight. Nuclei are stained with DAPI (blue), and Ester 488-labeled PSC small EVs are shown in green. Merged images indicate sEV uptake. Scale bar: 60 µm. **(F)** Quantification of Ester 488 fluorescence intensity in NDRG1-expressing MIAPaCa-2 cells compared to VC cells. Ester 488 fluorescence intensity is normalized to DAPI intensity. Each dot represents an independent experiment (n = 3), with bars indicating mean ± SEM. Statistical significance was determined using Student’s t-test (**p < 0.01).
