# Supplementary material for: Silencing the Signal: The Metastasis Suppressor NDRG1 Disrupts Small Extracellular Vesicle‐Mediated Crosstalk in Pancreatic Cancer
Source: J Extracell Vesicles. 2026 Jun 30;15(7):e70334. doi: 10.1002/jev2.70334 (PMC13317765; doi:10.1002/jev2.70334)
Supplement: Supplementary file 2 — Supporting Information: jev270334‐sup‐0001‐FigureS1–S5.docx [file JEV2-15-e70334-s001.pdf]

A.

MIAPaCa-2 VC Small EV

NANOSIGHT Capture 2025-02-13 10-50-54

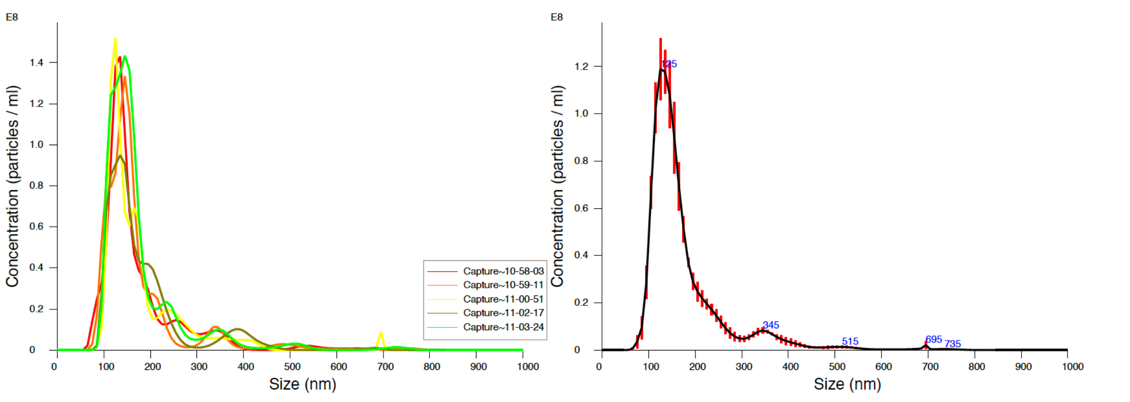

Stats: Mean +/- Standard Error

Metric	Value
Mean	175.7 ± 1.7 nm
Mode	136.0 ± 4.3 nm
SD	89.5 ± 1.3 nm
D10	98.8 ± 1.6 nm
D50	137.8 ± 1.7 nm
D90	266.0 ± 6.8 nm
**Concentration**	**1.06e+009 ± 5.30e+007 particles/ml**
Particles/frame	54.0 ± 2.7

MIAPaCa-2 NDRG1 Small EV

NANOSIGHT Capture 2025-02-13 11-30-07

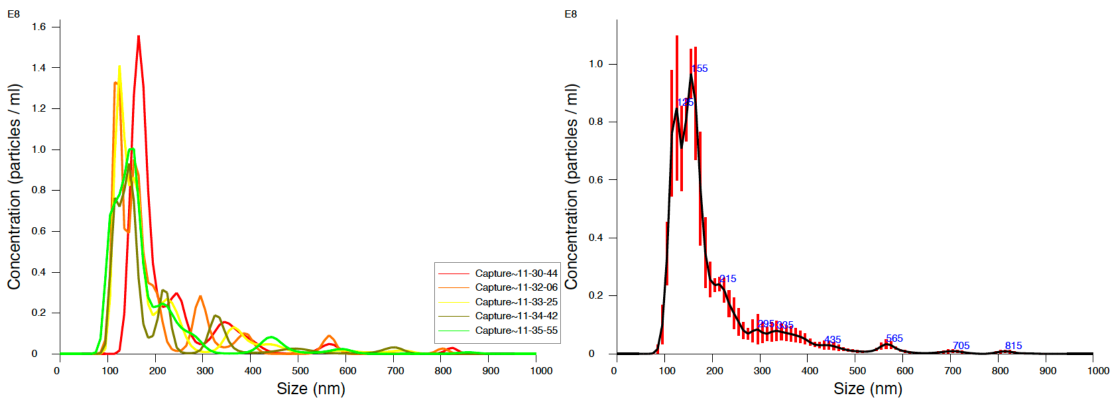

Stats: Mean +/- Standard Error

Metric	Value
Mean	197.9 ± 6.3 nm
Mode	141.0 ± 8.3 nm
SD	110.4 ± 2.7 nm
D10	110.6 ± 7.7 nm
D50	148.4 ± 5.3 nm
D90	319.8 ± 15.6 nm
**Concentration**	**9.27e+08 ± 4.99e+07 particles/ml**
Particles/frame	47.0 ± 2.5

B.

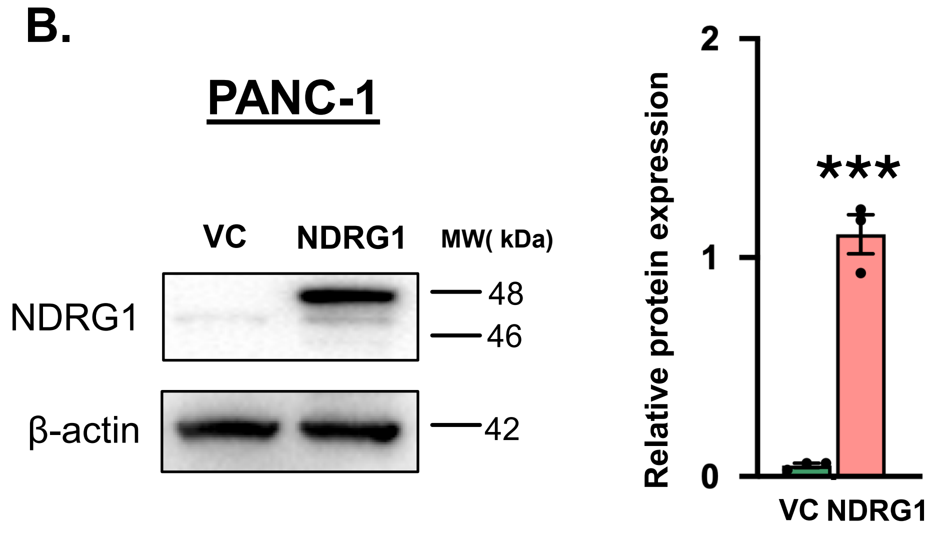

C.

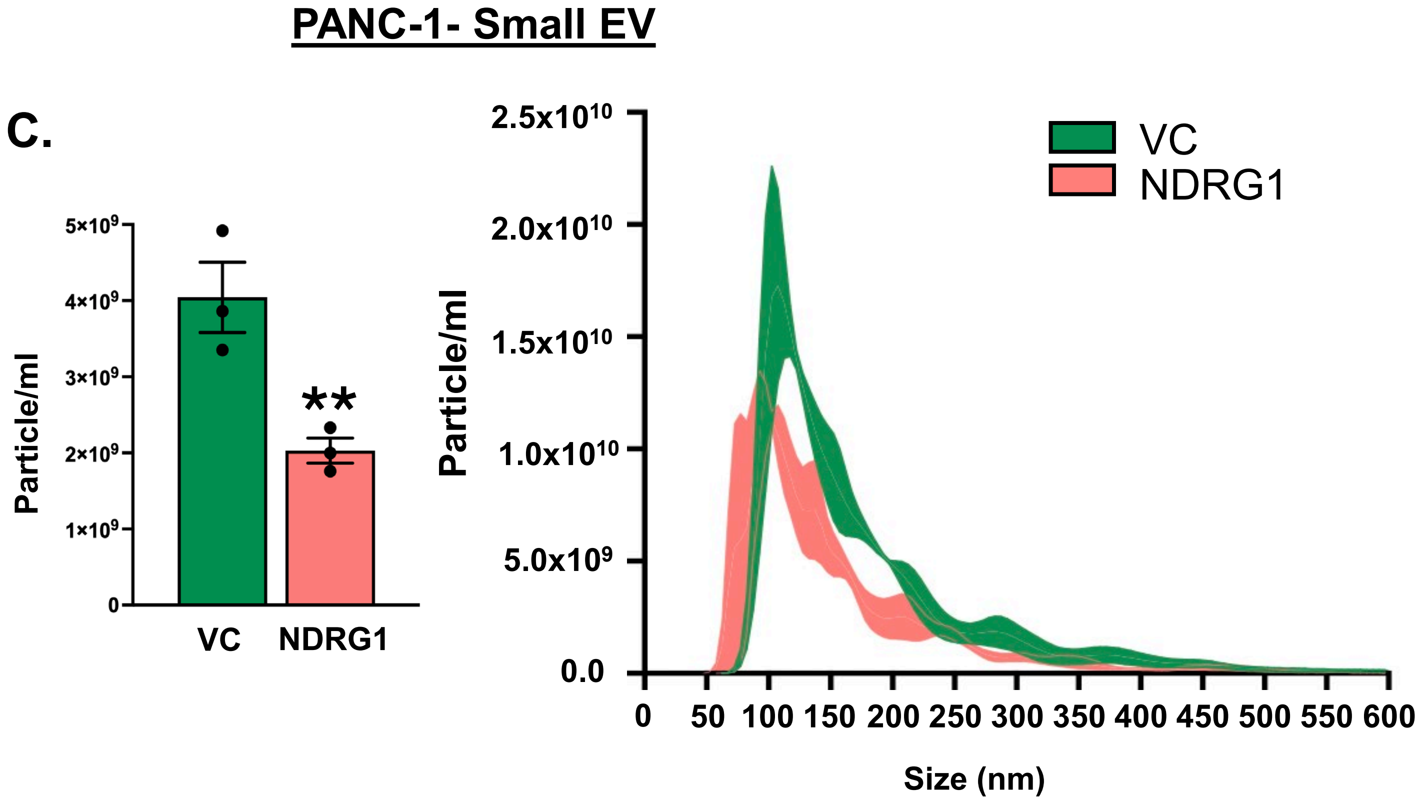

D.

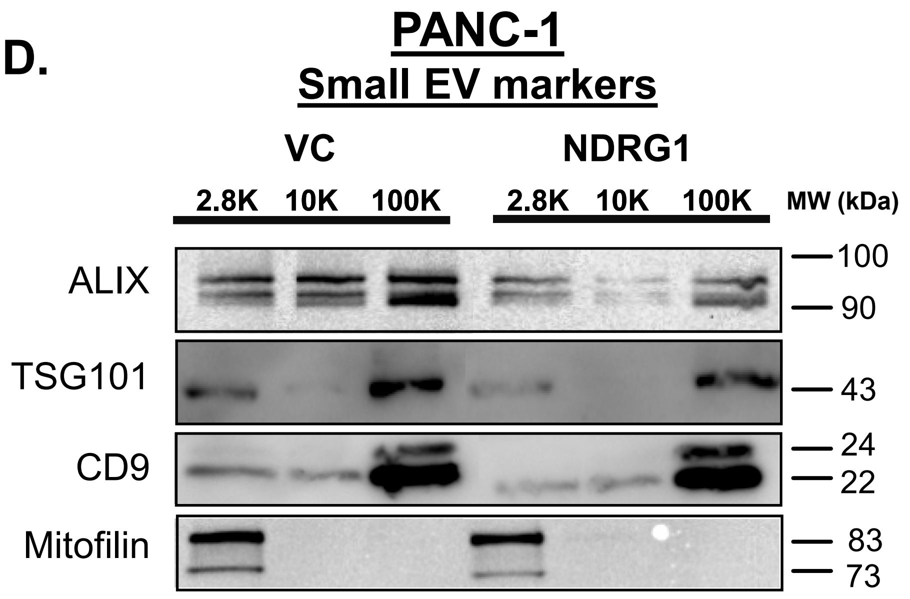

E.

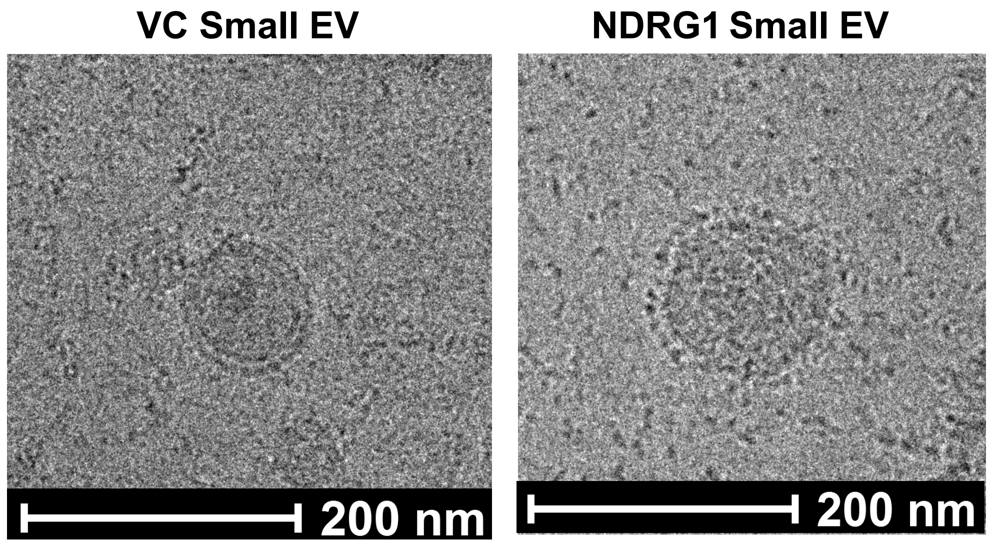

F.

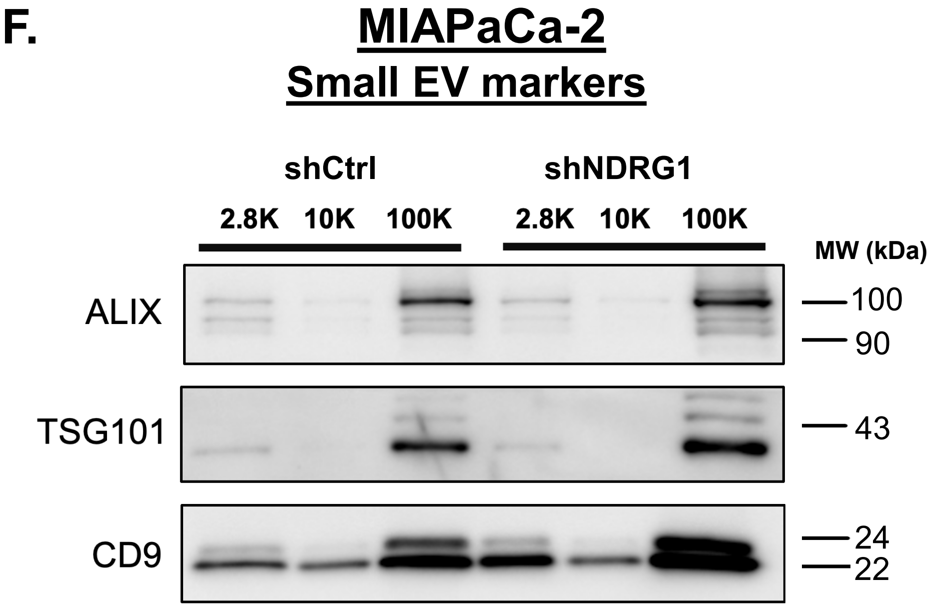

Supplemental Figure 1

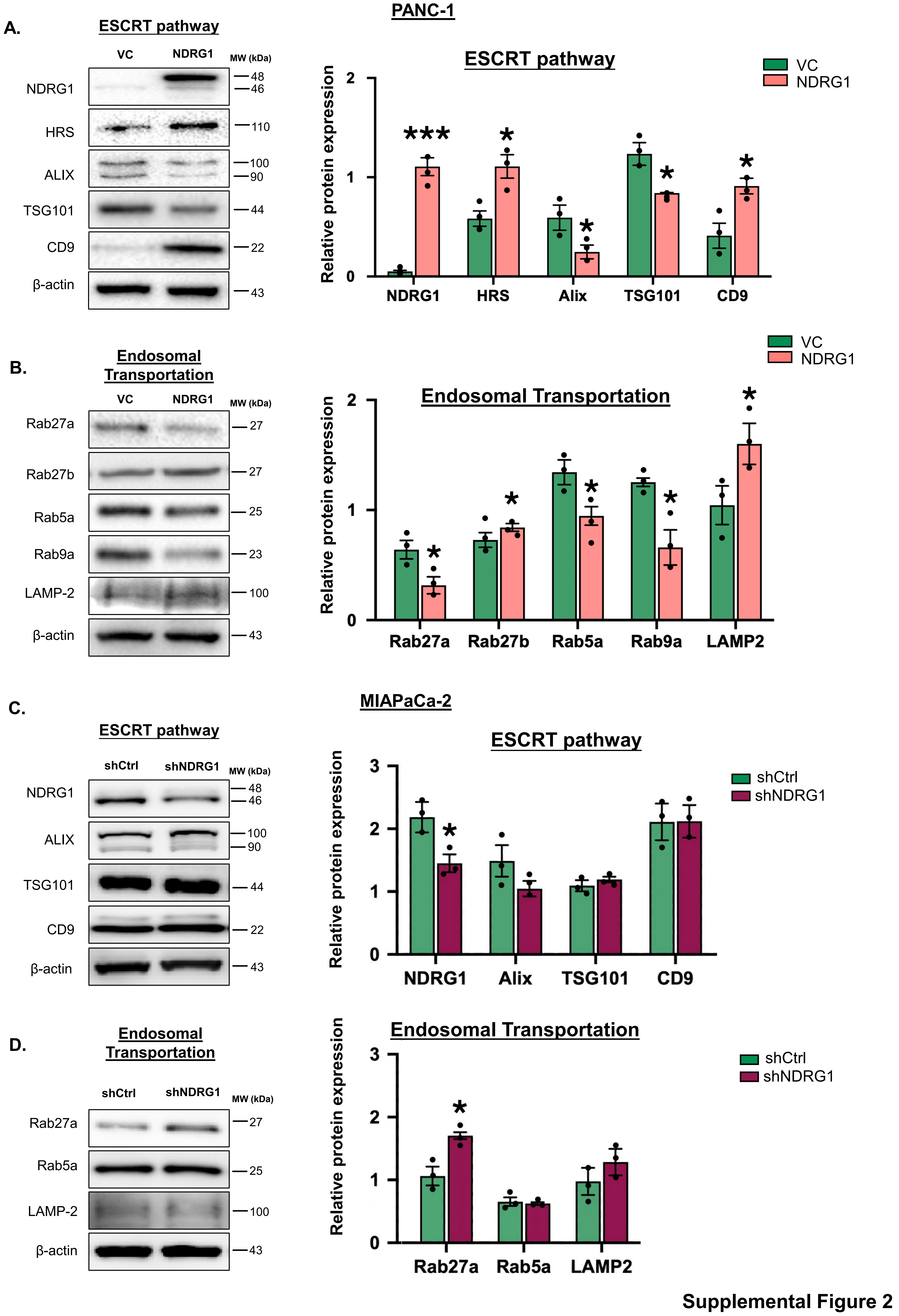
PANC-1
A.
ESCRT pathway
VC
NDRG1
MW (kDa)
NDRG1
HRS
ALIX
TSG101
CD9
β-actin
48
46
110
100
90
44
22
43
ESCRT pathway
Relative protein expression
VC
NDRG1
***
*
*
*
*
NDRG1
HRS
Alix
TSG101
CD9
B.
Endosomal Transportation
Rab27a
Rab27b
Rab5a
Rab9a
LAMP-2
27
25
23
Endosomal Transportation
Rab27a
Rab27b
Rab5a
Rab9a
LAMP2
MIAPaCa-2
C.
shCtrl
shNDRG1
D.
LAMP-2
Supplemental Figure 2

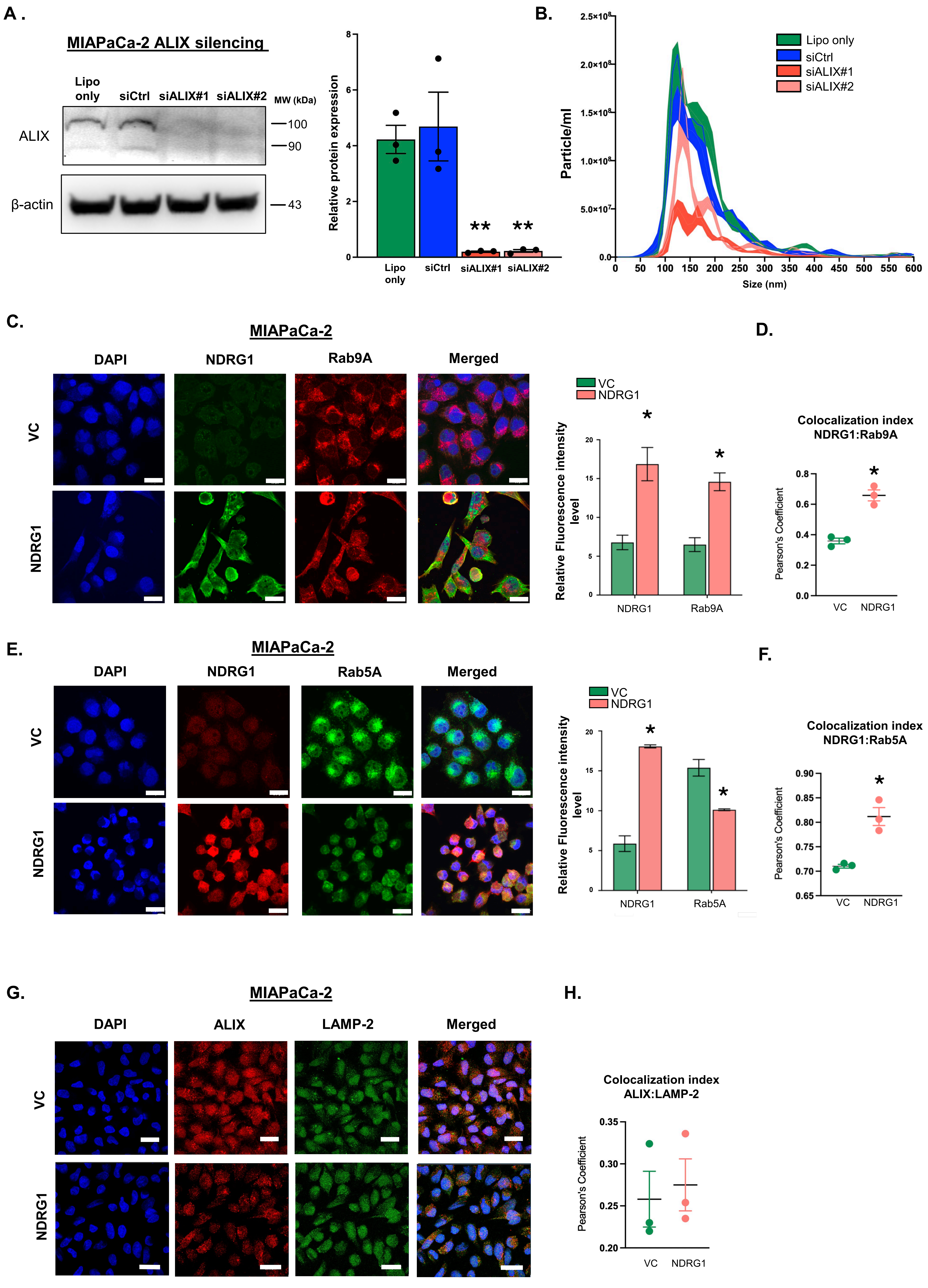

Supplemental Figure 3

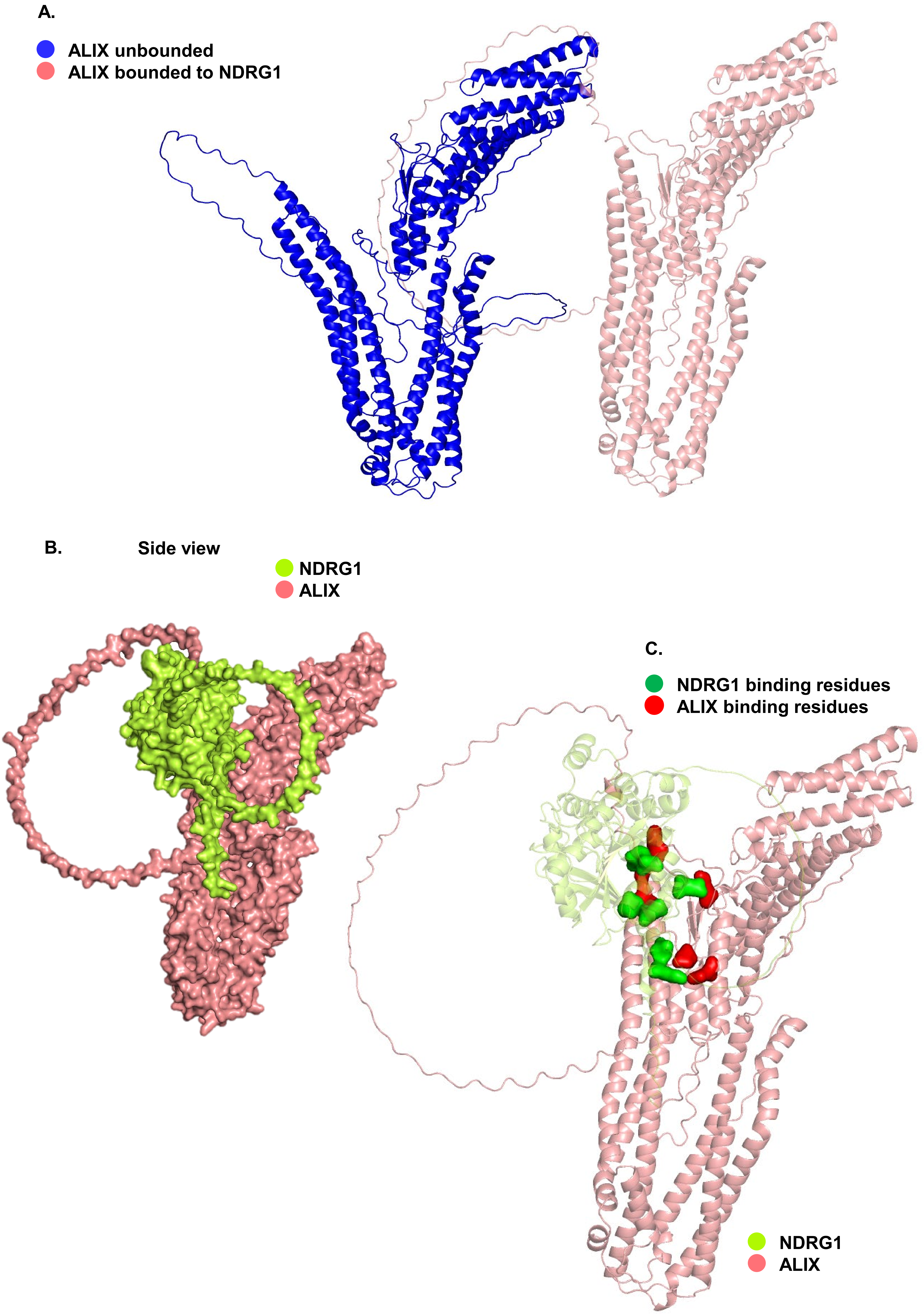

Supplemental Figure 4

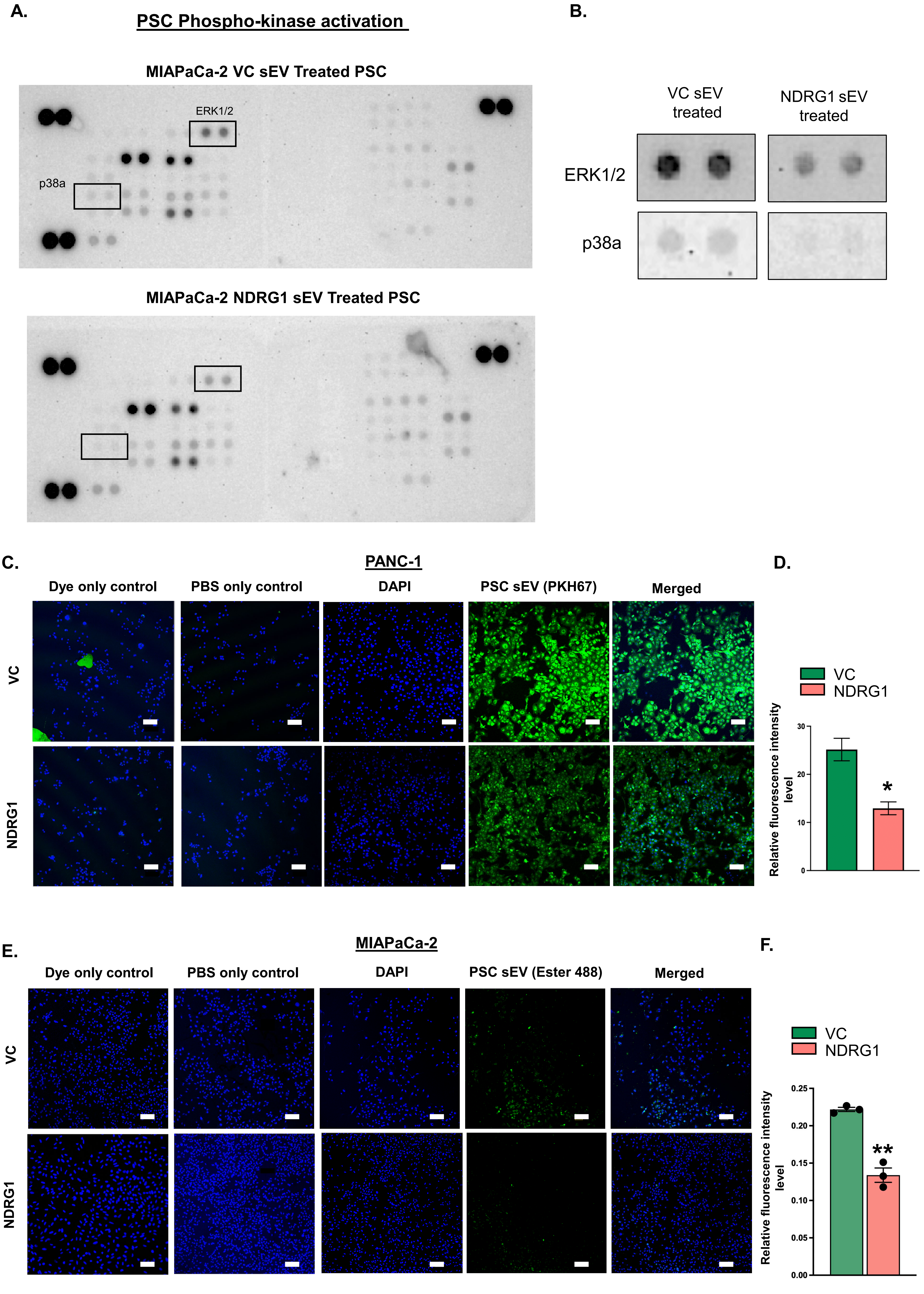

Supplemental Figure 5
